# *Lactobacillus rhamnosus* Sex-Specifically Attenuates Depressive-like Behavior and Mitigates Metabolic Consequences in Obesity

**DOI:** 10.1016/j.bpsgos.2023.02.011

**Published:** 2023-03-15

**Authors:** Mareike Schell, Kristina Wardelmann, Robert Hauffe, Michaela Rath, Simran Chopra, André Kleinridders

**Affiliations:** a German Institute of Human Nutrition Potsdam-Rehbruecke, Nuthetal, Germany; b German Center for Diabetes Research, Neuherberg, Germany; c Molecular and Experimental Nutritional Medicine, Institute of Nutritional Science, University of Potsdam, Nuthetal, Germany

**Keywords:** Depression, Dopamine, Emotionality, Insulin resistance, Obesity, Probiotics

## Abstract

**Background:**

Patients with diabetes exhibit an increased prevalence for emotional disorders compared with healthy humans, partially due to a shared pathogenesis including hormone resistance and inflammation, which is also linked to intestinal dysbiosis. The preventive intake of probiotic lactobacilli has been shown to improve dysbiosis along with mood and metabolism. Yet, a potential role of *Lactobacillus rhamnosus* (*Lacticaseibacillus rhamnosus* 0030) (LR) in improving emotional behavior in established obesity and the underlying mechanisms are unknown.

**Methods:**

Female and male C57BL/6N mice were fed a low-fat diet (10% kcal from fat) or high-fat diet (HFD) (45% kcal from fat) for 6 weeks, followed by daily oral gavage of vehicle or 1 × 10^8^ colony-forming units of LR, and assessment of anxiety- and depressive-like behavior. Cecal microbiota composition was analyzed using 16S ribosomal RNA sequencing, plasma and cerebrospinal fluid were collected for metabolomic analysis, and gene expression of different brain areas was assessed using reverse transcriptase quantitative polymerase chain reaction.

**Results:**

We observed that 12 weeks of HFD feeding induced hyperinsulinemia, which was attenuated by LR application only in female mice. On the contrary, HFD-fed male mice exhibited increased anxiety- and depressive-like behavior, where the latter was specifically attenuated by LR application, which was independent of metabolic changes. Furthermore, LR application restored the HFD-induced decrease of tyrosine hydroxylase, along with normalizing cholecystokinin gene expression in dopaminergic brain regions; both tyrosine hydroxylase and cholecystokinin are involved in signaling pathways impacting emotional disorders.

**Conclusions:**

Our data show that LR attenuates depressive-like behavior after established obesity, with changes in the dopaminergic system in male mice, and mitigates hyperinsulinemia in obese female mice.

SEE COMMENTARY ON PAGE 582

Across the globe, diabetes, obesity, and depression continue to increase. While obesity and diabetes are metabolic disorders, depression is a mental illness and a leading cause of disability that affects about 300 million people worldwide. Children who are obese at a young age have increased risk of developing lower self-esteem (1), and obesity often poses a weight stigma on people that is associated with psychological distress (2). Thus, there is a psychosocial burden of being obese (3), which might contribute to the association of metabolic and emotional disorders (4). While there are multiple causes for the association between obesity, diabetes, and emotional disorders, research progress over the last decade has established a potential common metabolic basis for these disorders. It has been proposed that depression and metabolic disorders share inflammation, oxidative stress, and hormone resistance due to mutually dysregulated signaling pathways. Obesity and diabetes are promoted in the presence of insulin resistance, which causes deteriorated glucose metabolism, hyperglycemia, and uncontrolled lipolysis. Disrupted brain insulin signaling, induced by genetic deletion of the insulin receptor using Nestin-Cre or GFAP-Cre mice, causes behavioral abnormalities with depressive-like behavior (5,6) and is linked to deteriorated dopaminergic signaling (7). The intake of a high-calorie, high-fat diet (HFD) with elevated levels of saturated long-chain fatty acids is sufficient to induce brain insulin resistance (8) and results in increased anxiety- and depressive-like behavior (9), disorders that are characterized by abnormal dopamine signaling. Interestingly, a HFD in rodents reduces the expression of tyrosine hydroxylase (TH), the rate-limiting enzyme in dopamine synthesis (10). Conversely, consuming a healthy diet is linked to mental health, a decreased prevalence of mental illnesses, and elevated TH levels (10,11).

The consumption of a HFD alters not only metabolism and behavior but also the gut microbiota composition (12). HFD-induced alterations of the microbiome are linked to emotional disorders and affect dopaminergic neurotransmission. Accordingly, feeding mice a HFD with an antibiotic cocktail, to erase an altered gut microbiota composition induced by HFD, attenuates anxiety- and depressive-like behavior (13).

Additionally, the use of prebiotics and probiotics to support a healthy microbiota composition has been tested to prevent the establishment of metabolic deteriorations and cognitive function (14). Lactobacilli represent prominent probiotics that are part of many dietary products that claim to be good for metabolism. The preventive supplementation of different *Lactobacillus* strains during obesity development can protect against metabolic dysfunction and improve mood (15,16). However, whether sex-specific differences exist or such a supplementation alters metabolism or behavior in established obesity has not been tested.

In this study, we investigated the effect of *Lactobacillus rhamnosus* (*Lacticaseibacillus rhamnosus* 0030) (LR) on emotional behavior in diet-induced obese mice. We show that a daily oral gavage for a minimum of 6 weeks reduces depressive-like behavior in male mice with established obesity. Moreover, this is linked to elevated gene expression of TH and a reversal of HFD-altered cholecystokinin (CCK) messenger RNA (mRNA) levels in the nucleus accumbens (NAcc). In female mice, HFD feeding causes hyperactivity, which can be mitigated by LR application. Furthermore, LR utilization attenuates HFD-induced hyperinsulinemia in female mice. Overall, our data show that the daily application of LR is able to reverse HFD-induced behavioral alterations in male mice, alters behavior-related gene expression, and further counteracts HFD-induced hyperinsulinemia in female mice.

## Methods and Materials

See the Supplement for the full description of the animal study and methods relating to metabolic and behavioral phenotyping, final procedures, and molecular and omics analyses.

### Animals

C57BL/6N female and male mice were obtained from Charles River Laboratories (Sulzfeld, Germany) and group housed in a temperature-controlled room (22 ± 1 °C) on a 12-hour light/dark cycle with free access to food and water. Mice were fed with either a semisynthetic low-fat diet (LFD) (10% of kcal from fat) or HFD (45% of kcal from fat) (both obtained from Research Diets, Inc.). From week 12 of age until the end of experiments, all mice received a daily peroral gavage of either 100 μL vehicle (phosphate-buffered saline [PBS]; Thermo Fisher Scientific Inc.) or 100 μL LR (*Lacticaseibacillus rhamnosus* 0030) (1 × 10^8^ colony-forming units of LR in PBS) to ensure accurate dosage.

### Behavior Tests

Behavioral assessment was performed weekly starting at week 14 of age. All tests were conducted during the light cycle. The tests were performed in the following order: open field test (OFT), light/dark box test (LDB), elevated plus maze test (EPM), and tail suspension test (TST). In a naïve cohort of male mice, tests were performed in the following order: TST, LDB, and splash test. An overview of the animal study is shown in Figure S1A.

### RNA Isolation and Reverse Transcriptase Quantitative Polymerase Chain Reaction

Total RNA was isolated from brain tissue using ReliaPrep RNA Tissue Miniprep System (Promega Corp.) according to manufacturer instructions. Following isolation, RNA was quantified by a NanoDrop spectrophotometer (Thermo Fisher Scientific Inc.) and reverse transcribed to complementary DNA using dNTP Set (Thermo Fisher Scientific Inc.), oligo(dT)_15_ primers (Promega Corp.), random hexamer primers (Promega Corp.), and M-MLV Reverse Transcriptase (Promega Corp.). Reverse transcriptase quantitative polymerase chain reaction was performed using GoTaq 1-Step RT-qPCR System mix (Promega Corp.) and 200 nM of each forward and reverse primer (obtained from MilliporeSigma) (see Table S1). Fluorescence was monitored using the ViiA7 Real-Time PCR System (Thermo Fisher Scientific Inc.). Each run was followed by a melt curve (90 °C to 60 °C) for quality control. Samples were analyzed in duplicate, and relative quantification of gene expression levels was performed according to the ΔΔCT method using TATA-box binding protein (*Tbp*) as reference gene. Data were expressed as 2^-ΔΔCT^ and relative to the respective control group, if not stated otherwise.

### Statistical Analysis

General statistical analysis was performed using GraphPad Prism 9 (GraphPad Software). Data are represented as mean ± SEM. For comparison of 2 groups, unpaired two-tailed Student’s *t* test was employed for parametric samples and Mann-Whitney *U* test was used for nonparametric samples. Comparison of multiple groups was performed by one-way analysis of variance (parametric samples) followed by Dunnett’s multiple comparison test to compare groups with a defined control group. To compare all groups with each other, Tukey’s multiple comparison test was used. For nonparametric samples, Kruskal-Wallis test and Dunn’s multiple comparison test were employed. The following *p* values indicate statistical significance: ∗*p* < .05, ∗∗*p* < .01, ∗∗∗*p* < .001, ∗∗∗∗*p* < .0001.

## Results

### Preventive Application of LR Counteracts HFD Feeding–Induced Reduction of TH Gene Expression

**Figure 1:**
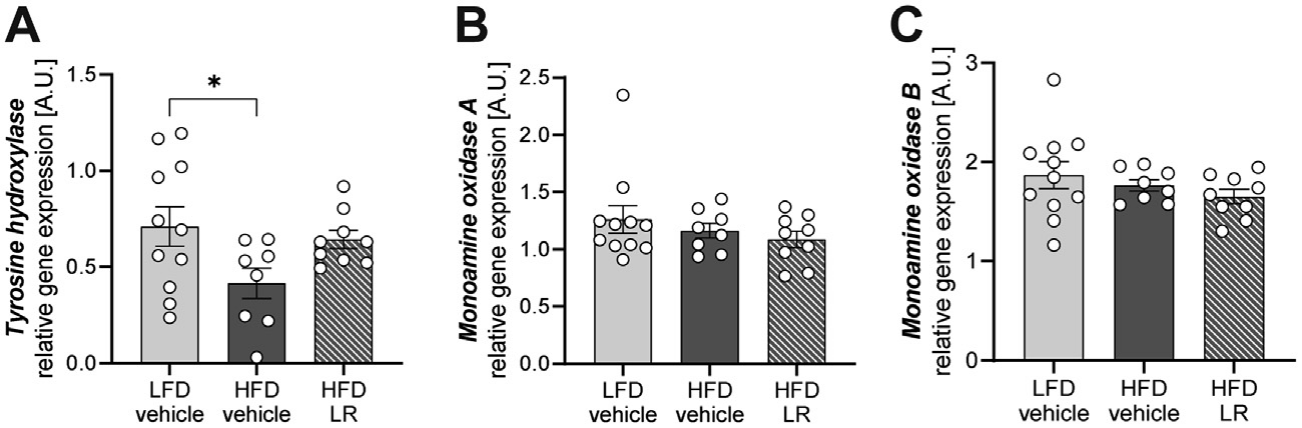
Preventive intervention with LR attenuates HFD-induced dysregulation of tyrosine hydroxylase in the caudate putamen. Relative messenger RNA levels of tyrosine hydroxylase **(A)** and monoamine oxidases A and B **(B, C)** in the caudate putamen of C57BL/6J male mice after 12 weeks of HFD. ∗*p* < .05 after one-way analysis of variance with Dunnett’s post hoc test (*n* = 8–11). All data are presented relative to *Tbp* (2^-ΔCT^) and as mean ± SEM. HFD, high-fat diet; LFD, low-fat diet; LR, *Lactobacillus rhamnosus*.

To determine whether diet-induced obesity and preventive application of probiotics can alter dopamine homeostasis, C57BL/6J male mice were fed a semisynthetic LFD or HFD for 12 weeks and, in parallel, received daily applications of either PBS (vehicle) as control or LR (now referred to as HFD LR). Interestingly, LR attenuated the HFD-induced reduction of the rate-limiting enzyme of dopamine synthesis in a dopaminergic brain region. While HFD feeding resulted in an approximately 42% decrease in TH mRNA levels in the striatum compared with LFD vehicle, HFD LR treatment attenuated this decrease, revealing indistinguishable gene expression levels of TH compared with LFD feeding (Figure 1A). Yet dopamine degrading enzymes monoaminoxidases A and B were unaffected (Figure 1B, C).

### HFD-Induced Metabolic Deteriorations in Female and Male Mice Before LR Application

Subsequently, we examined whether the LR application can counteract emotional alterations in established obesity conditions. Moreover, it is unclear whether behavioral alterations in obesity are caused by an overall excess of calorie intake or can be specifically linked to increased intake of lipids and fatty acids. To answer both questions, we fed male and female C57BL/6N mice a semisynthetic LFD as control and a HFD to establish diet-induced obesity.

After the initial 6 weeks of HFD feeding, female HFD-fed mice weighed more than LFD-fed control mice, exhibited a minor yet significant increase in blood glucose, and showed unaltered plasma insulin levels, while HFD-fed male mice exhibited increased body weight and hyperinsulinemia but no changes in blood glucose (Figures S2A–C, S4A–C). After randomization of HFD-fed mice, half of all HFD-fed mice received a daily oral gavage of LR for an additional 6 weeks, while the second group received PBS as control (HFD vehicle).

### LR Application After Established Obesity Reduces Plasma Insulin Levels in Female Mice but Does Not Alter Depressive-like Behavior

**Figure 2:**
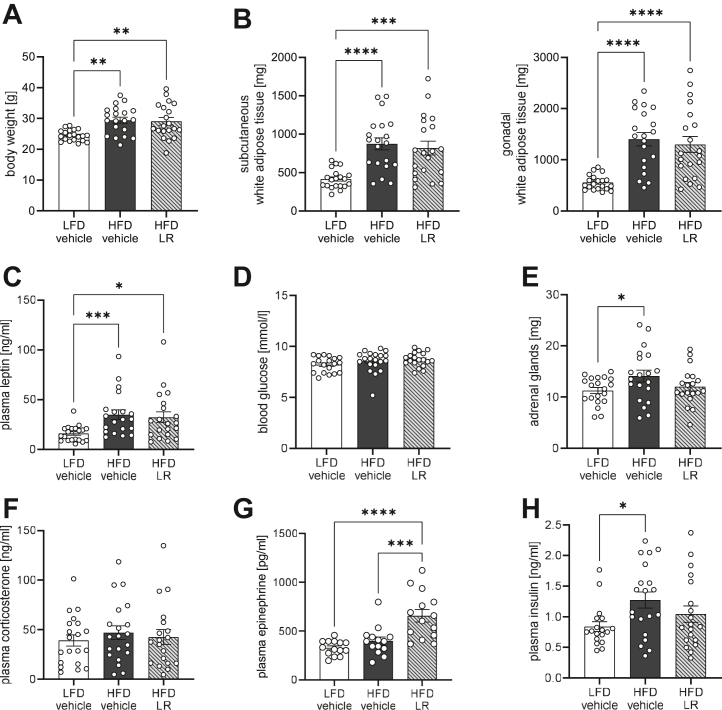
LR application regulates metabolism of obese female mice independent of changes in body weight. Body weight **(A)** and adipose tissue weights **(B)** of female mice after 13 weeks of HFD. **(C)** Plasma leptin levels of female mice after 10 weeks of HFD. Blood glucose levels **(D)** and adrenal glands weight **(E)** of female mice after 13 weeks of HFD. Plasma corticosterone **(F)**, plasma epinephrine **(G)**, and plasma insulin **(H)** levels of female mice after 10 weeks of HFD. ∗*p* < .05; ∗∗*p* < .01; ∗∗∗*p* < .001; ∗∗∗∗*p* < .0001 after one-way analysis of variance with Dunnett’s post hoc test **(A, B, E, G)** or after Kruskal-Wallis test with Dunn’s post hoc test **(C, H)** (*n* = 18–20). All data are presented as mean ± SEM. HFD, high-fat diet; LFD, low-fat diet; LR, *Lactobacillus rhamnosus*.

HFD feeding continued to increase body weight in female mice and caused elevated fat mass along with increased leptin levels compared with LFD feeding but did not alter other organ weights or blood glucose levels between all tested groups (Figure 2A–D and Figure S2D–F). In female mice, HFD feeding caused an increase in weight of adrenal glands compared with LFD mice, while LR application attenuated this increase, leading to similar adrenal gland weights as in LFD mice (Figure 2E). Yet, basal corticosterone levels were indistinguishable between all groups despite increased plasma epinephrine levels in LR-treated female mice (Figure 2F, G). In line with increased adrenal gland weight, HFD feeding resulted in elevated plasma insulin levels compared with LFD control. Again, the daily application of LR mitigated HFD-induced hyperinsulinemia, showing beneficial effects on insulin sensitivity (Figure 2H).

### Unaltered Depressive-like Behavior in LR-Treated Obese Female Mice

**Figure 3:**
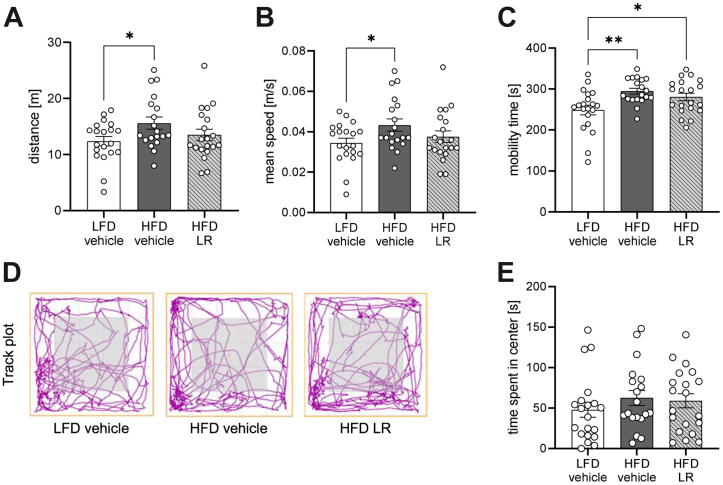
LR application regulates hyperactivity in diet-induced obese female mice. Distance **(A)**, mean speed **(B)**, and active time **(C)** of female mice after 8 weeks of HFD during the open field test. Exemplar track plots **(D)** and time spent in the center **(E)** of the field of female mice after 8 weeks of HFD during the open field test. Gray area represents the center of the field. ∗*p* < .05; ∗∗*p* < .01 after one-way analysis of variance with Dunnett’s post hoc test (*n* = 19–20). All data are presented as mean ± SEM. HFD, high-fat diet; LFD, low-fat diet; LR, *Lactobacillus rhamnosus*.

Next, we assessed emotional behavior in lean and obese mice as well as in obese mice treated with LR. First, we assessed exploratory behavior using an OFT in female mice that were fed a HFD for 8 weeks and received LR for 2 weeks. HFD feeding caused hyperactivity in female mice compared with LFD as determined by increased distance traveled, mean speed during the OFT, and overall mobility time (Figure 3A–C). LR application was able to mitigate HFD-induced increase in both distance and mean speed but did not affect overall mobility time. Interestingly, while locomotor activity was increased by HFD, exploration was unaffected by dietary interventions, as shown by an indistinguishable amount of time spent in the center of the field between tested groups (Figure 3D, E). Moreover, HFD feeding did not affect anxiety-like behavior as determined by the EPM and LDB, and it did not change depressive-like behavior, which was examined by the TST in female mice (Figure S3). Nevertheless, LR application increased the time spent in the open compartment during the EPM, indicating increased risk behavior (Figure S3B). Thus, HFD feeding induced hyperactivity in female mice, while LR treatment did not grossly alter emotional behavior in established HFD-induced obesity.

### LR Application Does Not Impact Metabolism in Obese Male Mice

**Figure 4:**
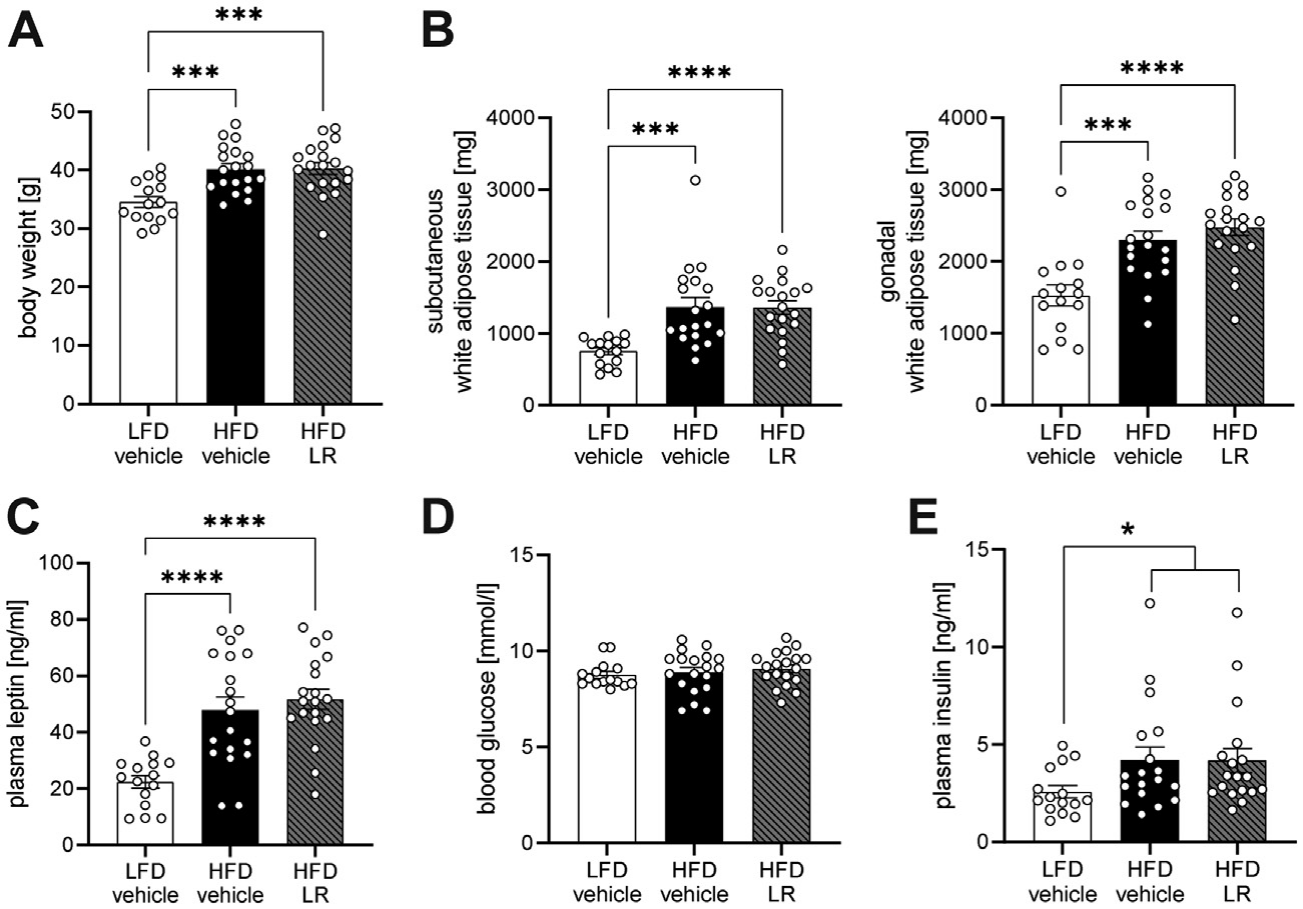
LR intervention does not modulate metabolism of obese male mice. Body weight **(A)** and white adipose tissue weights **(B)** of male mice after 13 weeks of HFD. **(C)** Plasma leptin levels of male mice after 10 weeks of HFD. **(D)** Blood glucose levels male mice after 13 weeks of HFD. **(E)** Plasma insulin levels of male mice after 10 weeks of HFD. ∗∗∗*p* < .001; ∗∗∗∗*p* < .0001 after one-way analysis of variance with Dunnett’s post hoc test **(A–D)** or after Kruskal-Wallis test with Dunn’s post hoc test for gonadal white adipose tissue **(B)** (*n* = 15–19). ∗*p* < .05 after Mann-Whitney *U* test **(E)**. All data are presented as mean ± SEM. HFD, high-fat diet; LFD, low-fat diet; LR, *Lactobacillus rhamnosus*.

Unexpectedly and in contrast to female mice, LR treatment did not attenuate or reverse any metabolic effects of HFD feeding in male mice, displaying indistinguishable elevated body weight, obesity, hyperleptinemia, and hyperinsulinemia, while also not changing blood glucose levels or organ weights compared with HFD male mice (Figure 4A–E and Figure S4D–G). However, similar to female mice, LR increased plasma epinephrine levels in male mice without affecting plasma corticosterone concentrations (Figure S4H, I). Additionally, LR treatment did not alter markers of inflammation in diet-induced obesity (Figure S4J).

### Depressive-like Behavior in Obese Male Mice Is Attenuated by LR Application

**Figure 5:**
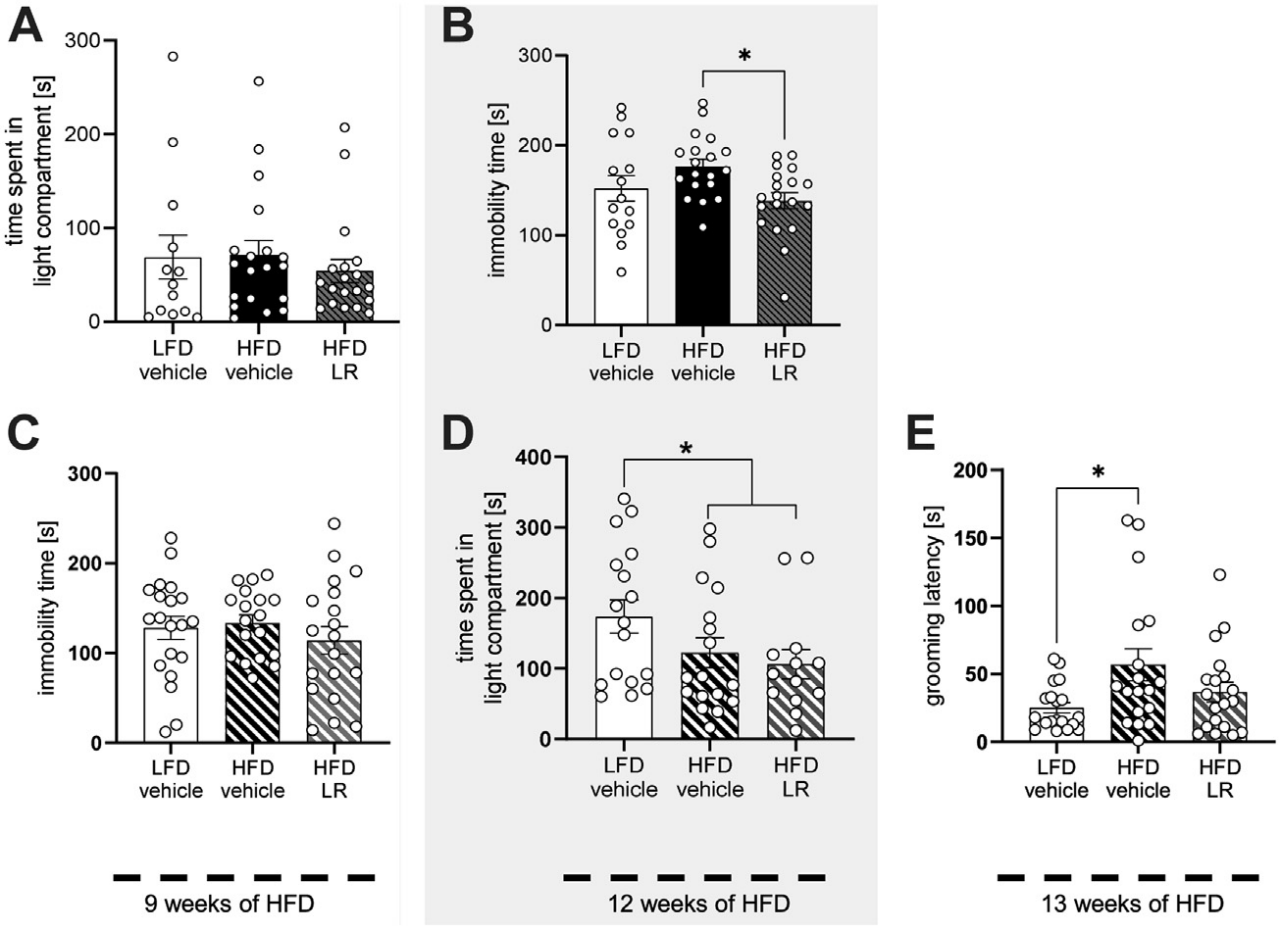
LR intervention alleviates aspects of HFD-induced depressive-like behavior but does not regulate anxiety. **(A)** Time spent in the light compartment of male mice after 9 weeks of HFD during the light/dark box test. **(B)** Immobility time of male mice after 12 weeks of HFD during the tail suspension test. **(C)** Immobility time of male mice after 9 weeks of HFD during the tail suspension test in a naïve cohort. **(D)** Time spent in the light compartment of male mice after 12 weeks of HFD during the light/dark box test in a naïve cohort. **(E)** Latency to initiate grooming of male mice after 13 weeks of HFD during the splash test in a naïve cohort. Gray area highlights behavior tests that were conducted in week 12 of HFD feeding. ∗*p* < .05 after one-way analysis of variance with Dunnett’s post hoc test **(B, E)** or after Mann-Whitney *U* test **(D)** (*n* = 13–20). All data are presented as mean ± SEM. HFD, high-fat diet; LFD, low-fat diet; LR, *Lactobacillus rhamnosus*.

In contrast to female mice, 2 weeks of LR application did not change activity during an OFT in HFD-fed male mice, and exploratory behavior also remained unaltered (Figure S5A–E). Furthermore, LFD vehicle, HFD vehicle, and HFD LR–fed mice did not differ significantly in the time they spent in the light compartment in the LDB, showing that LR did not alter anxiety-like behavior in this experimental setting (Figure 5A and Figure S5F). Similarly, stress-induced anxiety-like behavior was unaffected, as all groups traveled a similar distance and spent a similar amount of time in open arms during an elevated-X maze along with unaltered corticosterone levels after the test (Figure S5G–J).

Moreover, after 12 weeks of HFD feeding, LFD vehicle, HFD vehicle, and HFD LR–fed male mice differed significantly in their immobility time during a TST, as an indicator of altered emotional behavior. HFD-fed mice receiving daily oral gavage of LR for 6 weeks displayed reduced depressive-like behavior compared with HFD vehicle group, as assessed by an approximately 22% decrease in immobility time during the test (Figure 5B).

LR application affected depressive-like behavior but, unexpectedly, not anxiety (Figure 5A, B). As the LR treatment was 3 weeks shorter during the assessment of anxiety using the LDB compared with the TST, it raised the possibility that only a prolonged LR application was able to modulate anxiety-like behavior. Thus, anxiety- and depressive-like behavior were analyzed in a second naïve cohort in a reversed chronological order for the LDB and the TST. Feeding male mice a HFD for only 9 weeks with or without daily application of LR for 3 weeks revealed no differences in immobility time between all 3 tested groups (Figure 5C). After 6 weeks of daily LR application and 12 weeks of HFD feeding, only HFD feeding (combination of HFD and HFD LR groups) decreased the time that mice spent in the light compartment of the LDB, by approximately 33% compared with LFD vehicle group, indicative of HFD-induced anxiety. Yet, there was no difference in time spent in the light compartment between HFD vehicle and HFD LR male mice (Figure 5D).

To confirm that prolonged LR application was still able to alter depressive-like behavior in male mice, we further assessed the motivation of self-care during a splash test 1 week later. HFD feeding increased the time of grooming latency in mice compared with LFD control, as LR treatment of HFD-fed mice attenuated this phenotype resulting in a similar grooming latency as the LFD control mice, showing that LR treatment specifically improves motivation and reduces HFD-induced depressive-like behavior in male mice (Figure 5E).

### LR Treatment Exhibits Minor Effects on Microbiota Composition in Both Sexes

**Figure 6:**
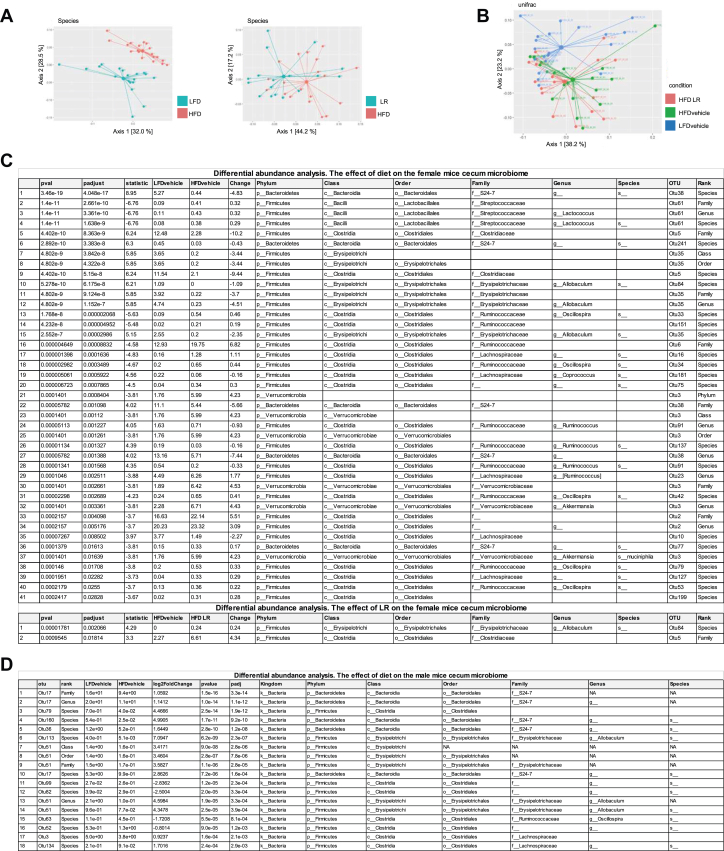
HFD intervention, but not LR application, influences cecal microbiota composition. **(A)** Principal component analysis plot of Unifrac distances on species level visualizing the effect of diet on the cecal microbiota beta diversity (*R*^2^ = 0.246, *p* = .001) and principal component analysis plot of Unifrac distances on species level visualizing the effect of LR on the cecal microbiota beta diversity (*R*^2^ = 0.093, *p* = .006) of female mice after 13 weeks of HFD. **(B)** Principal component analysis plot of Unifrac distances on species level visualizing the effect of diet (*R*^2^ = 0.18525, *p* = .028) and LR (*R*^2^ = 0.04523, *p* = .641) on the cecal microbiota beta diversity of male mice after 14 weeks of HFD. **(C)** Differential abundance analysis in female mice between LFD and HFD (diet effect) and HFD vs. LR (lactobacillus effect). **(D)** Differential abundance analysis in male mice between LFD and HFD (diet effect; ratio = LFD/HFD) and HFD vs. LR (lactobacillus effect; ratio = HFD/LR). **(C, D)** Adjusted *p* value < .05. Numbers within the graphs indicate individual animal identification numbers and correspond to individual samples. HFD, high-fat diet; LFD, low-fat diet; LR, *Lactobacillus rhamnosus*; NA, not available; OTU, operative taxonomic unit.

To gain insights into how HFD feeding and the therapeutic application of LR were able to specifically modulate emotional behavior, gut microbiota composition was determined in cecal samples using 16S ribosomal RNA gene amplicon sequencing, revealing only minor alterations in microbiota diversity. In female mice, diet (*p*val_Shannon_ = .016 on phylum level) and LR (*p*val_Shannon_ = .041 on phylum level) application caused minor yet significant changes in alpha diversity, while in male mice, alpha diversity remained unchanged (data not shown). Beta diversity revealed again a sexual dimorphism highlighting the importance of investigating both sexes. While HFD feeding significantly changed cecal microbiota composition in both sexes, LR modulated only beta diversity in female mice (Figure 6A, B). To get more insights into microbiota composition, we determined the differential abundances of operative taxonomic units (OTUs) in both sexes and between diets and treatment. In female mice, the abundance of 41 OTUs was significantly different due to HFD feeding. HFD predominantly increased the abundance of genera such as *Lactococcus*, *Oscillospira*, *Ruminococcus*, and *Akkermansia*, while the abundance of genera such as *Allobaculum* and *Coprococcus* was decreased. In contrast, LR changed the abundance of only 2 OTUs, with increased *Allobaculum* Otu84 abundance compared with the HFD group (Figure 6C). In male mice, only HFD feeding caused significantly different abundances of 18 OTUs, including the genera *Allobaculum* (which was decreased) and *Oscillospira* (which was increased) (Figure 6D).

### LR Application Alters the Metabolome in Plasma and Cerebrospinal Fluid in Obese Male Mice

**Figure 7:**
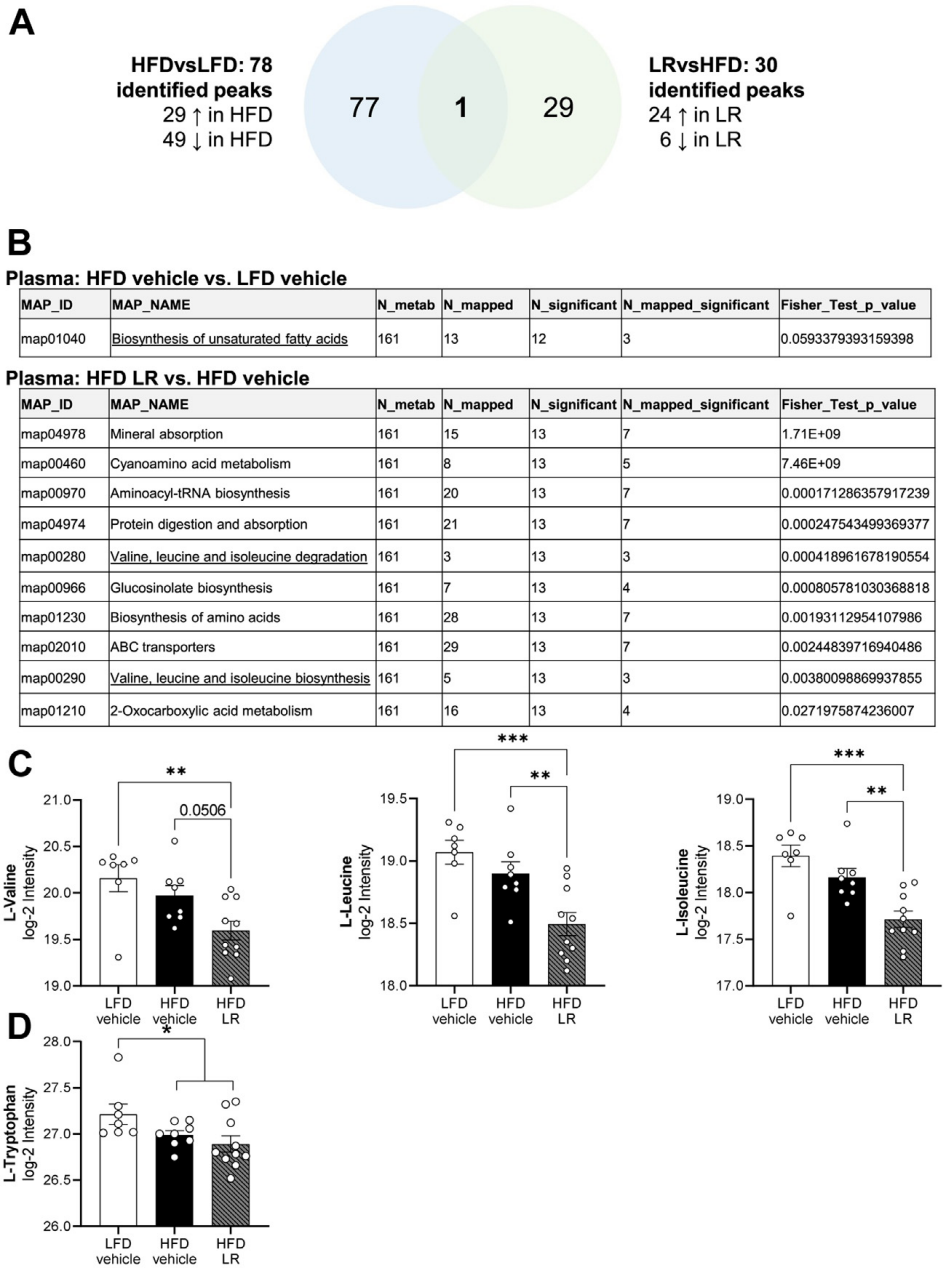
LR application, but not HFD, alters abundance of branched-chain amino acids in the plasma of male mice. **(A)** Visualization of significant annotated and nonannotated plasma metabolites within and between both comparisons (diet effect and lactobacillus effect) of male mice after 13 weeks of HFD. **(B)** Kyoto Encyclopedia of Genes and Genomes pathway analysis of annotated plasma metabolites and fatty acids of male mice after 13 weeks of HFD (adjusted *p* value < .05 after Fisher’s exact test). Relative abundance of L-valine, L-leucine, L-isoleucine **(C)**, and L-tryptophan **(D)** in plasma of male mice after 13 weeks of HFD. ∗*p* < .05; ∗∗*p* < .01; ∗∗∗*p* < .001 after one-way analysis of variance with Dunnett’s post hoc test **(C)** or unpaired two-tailed Student’s *t* test **(D)** (*n* = 7–10). All data are presented as mean ± SEM. ↑, significantly more abundant; ↓, significantly less abundant. HFD, high-fat diet; LFD, low-fat diet; LR, *Lactobacillus rhamnosus*; tRNA, transfer RNA.

As the abundance of *Allobaculum* has been linked to depressive-like behavior*,* altered metabolite concentrations, and neurotransmitters (17,18), we investigated only the metabolome of plasma and cerebrospinal fluid (CSF) samples from male mice. In the CSF, 44 metabolites (including lipid species) were significantly altered by a log2-fold change (log2FC) between HFD and LFD (diet effect) with 10 metabolites being upregulated and 34 being downregulated in HFD compared with LFD groups (Figure S6A). Additionally, 144 metabolites were significantly different between LR and HFD (lactobacillus effect), with 46 upregulated and 98 downregulated metabolites in the LR compared with HFD groups (Figure S6A). A similar result was observed in the plasma. Here, the abundance of 78 metabolites was found to be different between HFD and LFD, with 29 upregulated and 49 downregulated in HFD. Between LR and HFD, the abundance of only 30 metabolites was significantly different with 24 upregulated and 6 downregulated, and only one metabolite appeared in both comparisons that was not annotated (Figure 7A).

To gain more insights about overall regulation, a Kyoto Encyclopedia of Genes and Genomes (KEGG) pathway enrichment analysis of the CSF and plasma metabolome was performed only on annotated metabolites for the individual comparisons (HFD vs. LFD, LR vs. HFD) (*p* < .05). This analysis revealed that taste transduction (map04742) and glycerophospholipid metabolism (map00564) pathways were differentially regulated by diet or LR application in the CSF (Figure S6B). In contrast, HFD exposure predominantly altered metabolites in plasma that belong to fatty acid metabolism, whereas treatment with LR changed multiple pathways, including synthesis and degradation of branched-chain amino acids (BCAAs) (Figure 7B). Based on these results, we specifically determined the relative abundance of single BCAAs in CSF and plasma. While diet compared with LR treatment had a stronger effect on BCAAs in CSF (Figure S6C), LR was the driving factor for reduced peripheral BCAA levels without altering *Slc7a5* gene expression for BCAA uptake (Figure 7C, Figure S6D). Although plasma serotonin was not detected in our unbiased metabolomic approach, the abundance of its precursor L-tryptophan was decreased in HFD feeding, suggesting a modulation of serotonin production (Figure 7D, Figure S7A). Overall, HFD and LR application caused mild alterations in serotonergic-related metabolites in male mice (Figure S7A, B).

### Complex Gene Regulation of Key Enzymes of Neurotransmitter Signaling Affecting Behavior by LR Application in Obese Male Mice

**Figure 8:**
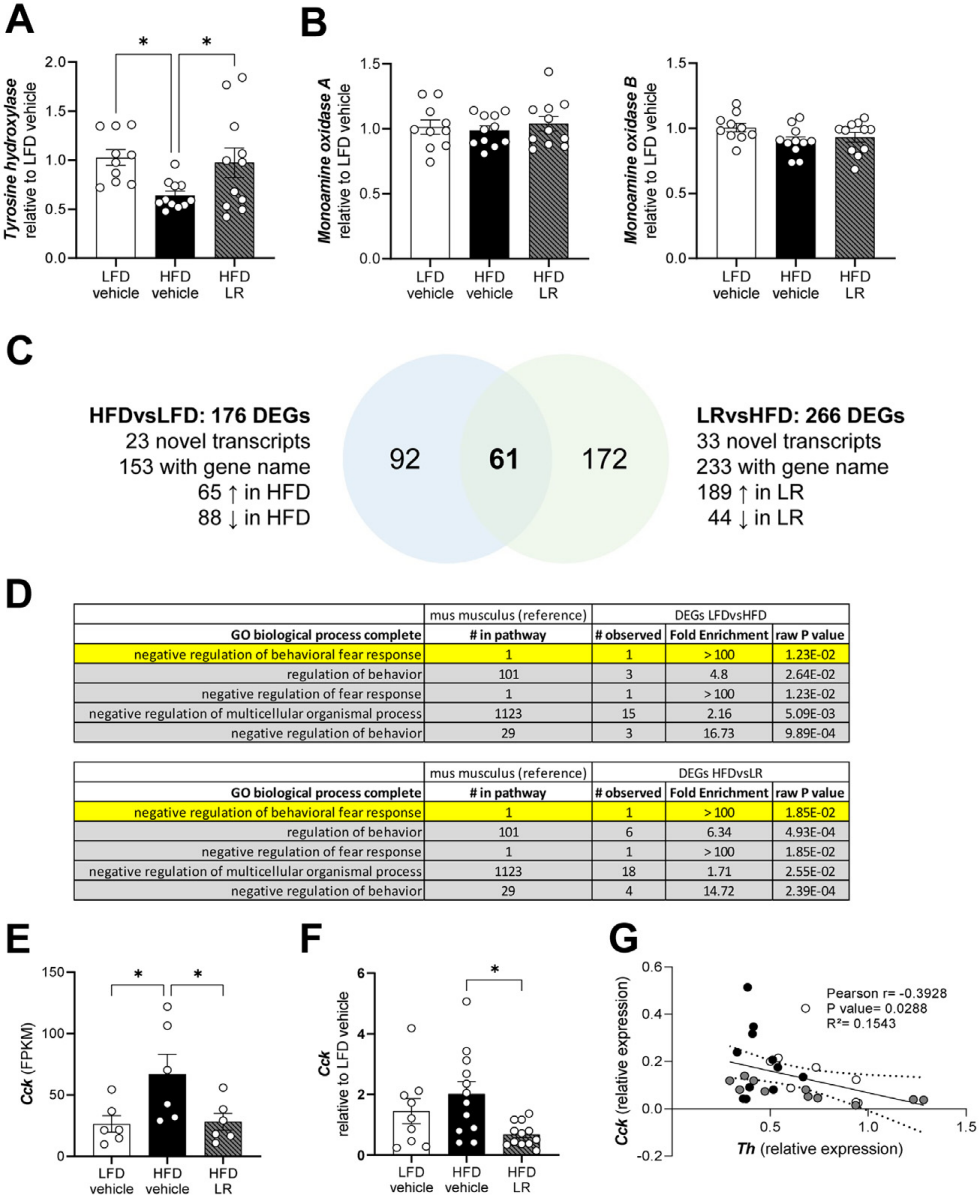
LR attenuates HFD reduction in tyrosine hydroxylase gene expression in the nucleus accumbens and reveals a signature of altered cholecystokinin expression. Messenger RNA expression of tyrosine hydroxylase **(A)** and monoamine oxidases A and B **(B)** in the nucleus accumbens of male mice after 13 weeks of HFD using reverse transcriptase quantitative polymerase chain reaction. **(C)** Visualization of significant (*p* < .05) DEGs within and between both comparisons (diet effect and lactobacillus effect) in the nucleus accumbens of male mice after 13 weeks of HFD (*n* = 6). **(D)** Significantly enriched biological processes (GO) for all annotated genes of DEGs within and between both comparisons (diet effect and lactobacillus effect) of male mice after 13 weeks of HFD using Fisher’s exact test and no correction for multiple testing (raw *p* value). Cholecystokinin messenger RNA levels using RNA sequencing **(E)** and reverse transcriptase quantitative polymerase chain reaction **(F)** for validation. **(G)** Correlation analysis of relative gene expression of *Th* and *Cck*. All data are presented relative to *Tbp* (2^-ΔCT^). The continuous line represents the mean and the dotted line represents the error after linear regression analysis. ∗*p* < .05 after one-way analysis of variance with Dunnett’s post hoc test **(A, E, F)** or Pearson correlation **(G)**. All data are presented as mean ± SEM. DEGs, differentially expressed genes; FPKM, fragments per kilobase of transcript per million mapped reads; GO, Gene Ontology; HFD, high-fat diet; LFD, low-fat diet; LR, *Lactobacillus rhamnosus*.

As diet-induced emotional alterations are also affected by the dopaminergic system and especially by dopamine metabolizing enzymes (10), we further analyzed gene expression of key enzymes in the caudate putamen (striatum), ventral tegmental area/substantia nigra, and NAcc. HFD exposure did not affect gene expression of TH or monoamine oxidase A and B in samples of caudate putamen as well as ventral tegmental area/substantia nigra from male mice (Figure S8A, B). Importantly, HFD decreased TH gene expression by approximately 38% compared with LFD in the NAcc (Figure 8A). Again, mRNA levels of monoamine oxidase A and B along with gene expression of dopamine transporter were not regulated (Figure 8B, Figure S8C). The HFD-induced decrease of TH mRNA levels was reversed by LR treatment in NAcc (Figure 8A), but this result could not be confirmed on the protein level using Western blot (Figure S8D) and immunohistochemistry analysis (Figure S8E). There was no direct correlation between *Th* mRNA levels and immobility time or body weight (Figure S9A–C). As tryptophan hydroxylase mRNA level, a marker for serotonergic metabolism, was also unaltered (Figure S9D–F), our data suggest that additional factors might contribute to this behavioral phenotype.

To gain a better understanding of HFD and LR-induced alterations in gene expression patterns of the dopaminergic system, we further performed RNA sequencing analysis on NAcc samples of the different groups. The samples were matched and selected according to their variability in gene expression levels of TH, resulting in the same HFD-induced reduction by approximately 36% compared with LFD control and displaying a similar variation in the LR group (*n* = 6 samples per group). HFD compared with LFD led to 176 differentially expressed genes (DEGs) ([log2FC]>|1.0|; raw *p* < .05) (Figure 8C). Unexpectedly, TH was not found among those DEGs because of a [log2FC]<|1.0| (HFD vs. LFD [log2FC] = −0.6021 and LR vs. HFD [log2FC] = 0.5939). Nevertheless, TH mRNA levels were significantly decreased by HFD feeding (*p* = .0034 after unpaired two-tailed Student *t* test), and there was a strong trend toward increased TH gene expression by LR application (*p* = .0942 after unpaired two-tailed Student *t* test) (Figure S10A). Of the 176 differentially regulated genes between HFD and LFD, 153 had an annotated gene name, with 65 genes being upregulated in the HFD-fed group and 88 genes being downregulated in HFD (Figure 8C).

To evaluate the effect of LR application in this context, DEGs between LR and HFD were determined. In total, 266 DEGs were identified, of which 233 had an annotated gene name. Within those 233 DEGs, 189 were upregulated in the LR-treated group, while 44 were downregulated. Interestingly, 61 DEGs overlapped in both comparisons and were subsequently evaluated using a heatmap by plotting the respective log2FC (Figure 8C). Interestingly, all genes that were significantly downregulated by HFD feeding (compared with the LFD group) were significantly upregulated due to LR application (compared with HFD vehicle) and vice versa, suggesting a rescue effect by LR on gene expression level in HFD-fed male mice (Figure S10B). Of 61 DEGs, 58 were successfully mapped to proteins (STRING protein network), and subsequent network analysis revealed altered regulation of neuropeptide hormone activity (GO:0005184), neuropeptide signaling pathway and peptide hormone binding (CL:19959), and neuroactive ligand-receptor interaction (mmu04080) (Figure S10C). To confirm this finding, gene names of DEGs between LFD and HFD as well as between HFD and LR were reanalyzed using Gene Ontology Enrichment Analysis of the PANTHER Classification System, which revealed significant modulation of pathways involved in the regulation of behavior (Figure 8D). CCK was one of 5 genes that were involved in the regulation of behavior and showed a significant upregulation of CCK gene expression in the HFD vehicle group compared with LFD (Figure 8E). Interestingly, while *Cck* was upregulated in diet-induced obesity, LR application in HFD-fed mice normalized *Cck* levels (Figure 8E). Further, *Cckbr*, but not *Cckar*, expression was significantly increased in HFD-fed mice, which was not observed in mice receiving LR application (Figure S10D). Strikingly, we confirmed the dysregulation of *Cck* using a targeted quantitative polymerase chain reaction approach between tested groups with significantly reduced CCK gene expression due to LR application in HFD-induced obesity (Figure 8F). Lastly, correlation analysis revealed a significant negative correlation between relative expression of *Th* and *Cck* (Figure 8G) (19).

In summary, LR prevented HFD-induced increase of plasma insulin and adrenal gland weight in C57BL/6N female mice with established obesity and modulated HFD-induced hyperactivity. In contrast, LR did not exert metabolic effects in diet-induced obese male mice but instead specifically regulated depressive-like and goal-directed behavior with altered TH and CCK gene expression levels in the NAcc, which are potential mediators of LR-induced attenuation of HFD-induced emotional alterations.

## Discussion

The intake of an unhealthy high-calorie diet causes dysbiosis and is linked to altered emotional behavior in humans (20). As this dietary intake also impacts the microbiome, preventive supplementation of probiotics has been shown to improve gut health, metabolism, and emotional behavior in rodents. Yet, it is not well understood how efficient such an intervention in established obesity might be and how this influences the fat content and the quality of fat in this context. In this study, we show that the increased content of unhealthy fat (lard) in the diet is sufficient to cause hyperactivity in female mice and depressive-like behavior in male mice, while supplementation of LR ameliorates HFD-induced depressive-like behavior in male mice and attenuates elevated insulin levels in diet-induced obesity in female mice.

To make arguments about the quality of fat, we used a semisynthetic LFD containing 10% calories from fat as a control in our study. Both diets (LFD and HFD) contained the same amount of sucrose and soybean oil, while the HFD (45% calories from fat) contained a higher amount of lard, indicating that the observed effects were not only dependent on a difference in fat (Δ fat = 35%) but also a difference in lard content, which is rich in, e.g., long-chain saturated fatty acids and ω-6 polyunsaturated fatty acids. Palmitate has been shown to be especially detrimental for brain insulin sensitivity (8), whereas linoleic acid causes greater weight gain than saturated fatty acids and impairs glucose metabolism in male mice (21). Thus, the observed impact on metabolism and emotional behavior can be attributed to a difference in fat content. This is important, as the percentage of calories from fat along with different fat sources can exert different behavioral and metabolic effects. It has been shown that different dietary fat types cause specific alterations in anxiety- and depressive-like behavior (22) as well as in insulin sensitivity (23). Thus, the moderate effect of feeding a 45% HFD on basic metabolic parameters and insulin sensitivity compared with a semisynthetic LFD accounts for the mild effect on emotional behavior. Furthermore, biological sex influences emotional behavior in mice (24), explaining the observed differences in emotional behavior between male and female mice in our study.

It had been shown that preventive *Lactobacillus* strain supplementation can attenuate the development of obesity, insulin resistance (15,25), and emotional alterations (26). While we show that in established obesity, LR supplementation is able to improve depressive-like behavior in male mice, HFD LR–fed female mice exhibited unaltered insulinemia and adrenal glands weight compared with LFD-fed mice, thus alleviating the detrimental effect of HFD-induced hyperinsulinemia. Reasons for this sex difference are so far unknown.

Although HFD feeding exhibited an increase in adrenal gland mass in female mice, mice of both sexes showed elevated plasma epinephrine levels exclusively in the HFD LR group without affecting plasma corticosterone or blood glucose levels. Besides its classical role in stress signaling, epinephrine has been shown to improve cognitive function, memory for emotionally arousing experiences, and depression as well as motivation (27, 28, 29). Along with an influence on the dopaminergic system (30), this might offer an explanation for the observed decrease in depressive-like behavior in male mice, as both performed tests partially rely on differences in motivation and dopaminergic signaling (31,32) (Figure 5B, E).

In male mice, LR treatment did not affect insulin sensitivity in obesity, but it did ameliorate depressive-like behavior. This is in contrast to the preventive application of lactobacillus in mice, which attenuates the development of diet-induced obesity with insulin resistance (15,33,34). In established obesity, LR treatment did not alter markers of inflammation in diet-induced obesity (Figure S4J), suggesting that the reversal of inflammation is needed to improve metabolism in male mice.

Another reason for the observed differences in modulating either behavior or metabolism relates to the duration of administration (diet and/or LR) in our experimental setting. Prolonged probiotic administration exerts more profound effects on health (35, 36, 37). A time-dependent effect is also known from drugs used to treat psychiatric diseases. These drugs need to reach a certain threshold to induce their therapeutic effect (38). Similar data about differences in behavioral outcomes can also be observed for dietary interventions. A transient effect of HFD feeding on exploration in male mice has been observed only after 3 weeks (39). In our study, after 8 weeks of HFD, activity and exploration remained unchanged, which was also observed after 10 weeks of HFD during the EPM. Interestingly, HFD feeding affected anxiety- and depressive-like behavior only after 12 (LDB and TST) and 13 (splash test) weeks, indicating that a long-term exposure is necessary to disturb behavior. Similarly, male mice receiving a 45% HFD for a minimum of 3 months exhibited changes in locomotion, while it took at least 5 months of HFD feeding to decrease exploration and increase anxiety-like behavior (18).

An unexpected observation of our study represented the specific attenuation of depressive-like behavior by LR in male mice while anxiety-like behavior was unaffected (Figure 5). Anxiety- and depressive-like behavior share common dysregulated pathways and are often observed in the same models (5,9). Elevated CCK in the brain as well as the administration of CCK receptor agonists has been shown to deteriorate emotional behavior by altering dopaminergic signaling (40,41). We have identified a specific genetic signature of alterations in dopaminergic and CCK signaling indicating that the presence of alterations in both pathways modulates depressive-like, but not anxiety-like, behavior. Interaction between these signaling pathways is affected in depression, and their involvement can contribute to depressive-like behaviors (42). Moreover, elevated central CCK signaling can suppress dopaminergic signaling in addition to TH activity and impact emotional behavior (19,43,44). Lastly, on a molecular level, palmitate has been shown to induce CCK signaling in hypothalamic neurons, connecting the exposure to a lard-based HFD to elevated CCK in the brain (45). Moreover, a decrease in TH and low levels of dopamine due to increased turnover have previously been associated with alterations in emotional behavior (5,10), and we confirmed this observation, showing that TH mRNA levels were decreased in HFD conditions (RNA sequencing and quantitative polymerase chain reaction) as a sign of altered dopaminergic signaling. Why TH protein levels were unaffected in this scenario remains unknown, but it can be explained by spatial differences in transcription and translation as well as regulation of the catalytic activity by phosphorylation (46) [reviewed in (47,48)].

While HFD-fed male mice usually respond with a decrease in activity, diet-induced obese female mice display hyperactivity (49,50). Interestingly, the increase in physical activity, as observed in female mice fed a HFD, can be mediated by the mesolimbic pathway, where estrogen stimulates dopamine release in the NAcc, supporting the potential presence of differentially affected dopamine signaling in both sexes. Conversely, inhibition of dopaminergic signaling decreases physical activity (51).

Only minor changes in cecal microbiome composition were detected in female and male mice; however, both HFD feeding and LR application had an effect on beta diversity in female mice (Figure 6C), while male mice exhibited alterations only in response to the HFD. Analysis of differential abundances shows that *Allobaculum* is linked to metabolic and behavioral alterations in female mice (Figure 6E). Here, HFD feeding decreased abundance of *Allobaculum*, which was increased after LR treatment. Interestingly, reduced abundance of *Allobaculum* is linked to chronic stress and depressive-like behavior (17,18), confirming the association of an altered abundance of the genus *Allobaculum* to emotional disorders. On the other hand, elevated *Allobaculum* abundance is linked to improved insulin signaling (52) (Figure 2H). The differences in abundance of *Allobaculum* can be explained by the decreased amount of carbohydrates in HFDs, as *Allobaculum* grows in the presence of exopolysaccharides (53), which can be synthesized by LR (54). Increased *Allobaculum* abundance is linked to BCAA catabolism (55) and thus connects our observed alteration in microbiota to altered BCAA metabolism, as seen in our Kyoto Encyclopedia of Genes and Genomes analysis (Figure 7B,C). Interestingly, a reduction of plasma BCAA levels in HFD-fed mice has been shown to specifically improve depressive-like behavior via serotonergic signaling (56), which supports the presence of altered emotional behavior.

Taken together, our data show that LR application exhibits sex-specific effects in established obesity. While metabolism is positively affected in obese female mice, male mice exhibit improved emotional behavior.

## Supplementary Material

Supplementary Material

Key Resources Table
