# Supplementary material for: Lactobacillus rhamnosus Sex-Specifically Attenuates Depressive-like Behavior and Mitigates Metabolic Consequences in Obesity
Source: Biol Psychiatry Glob Open Sci. 2023 Mar 15;3(4):651–62. doi: 10.1016/j.bpsgos.2023.02.011 (PMC10593880; doi:10.1016/j.bpsgos.2023.02.011)
Supplement: Supplementary Material [file mmc2.pdf]

SUPPLEMENTARY INFORMATION

Lactobacillus rhamnosus Sex-specifically Attenuates Depressive-like Behavior and Mitigates Metabolic Consequences in Obesity

Schell *et al.*

Supplementary figures

A

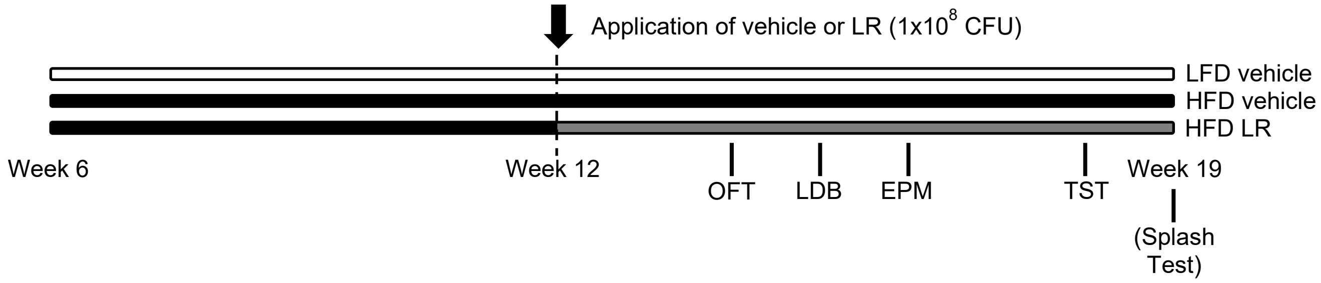

Suppl. Figure 1: Additional metabolic data of female mice. (A) Overview of the animal study design. Detailed description can be found in the Supplementary methods. LR: *Lacticaseibacillus rhamnosus* 0030, OFT: Open Field Test, LDB: Light/Dark Box Test, EPM: Elevated Plus Maze, TST: Tail Suspension Test, LFD: low-fat diet, HFD: high-fat diet.

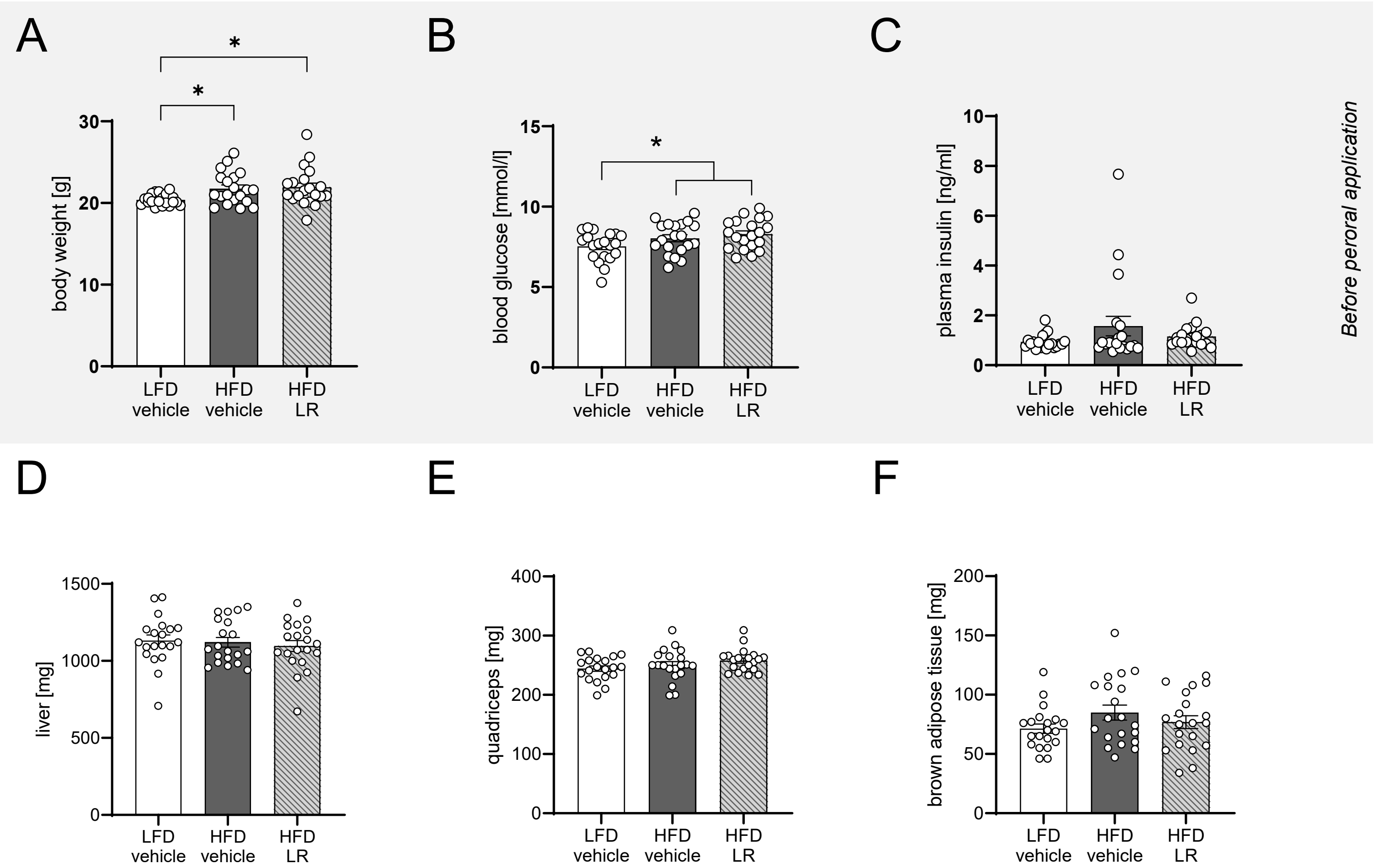

Suppl. Figure 2: Additional metabolic data of female mice. (A) Body weight, **(B)** blood glucose, and **(C)** plasma insulin of female mice after 6 weeks of HFD before LR application. **(D)** Liver weight, **(E)** quadriceps weight, and **(F)** brown adipose tissue weight of female mice after 13 weeks of HFD. * P<0.05 after 1-way ANOVA with Dunnett's Post-hoc test (A) or after unpaired two-tailed Student's t-test (B) (n= 20). All data are presented as mean±SEM.

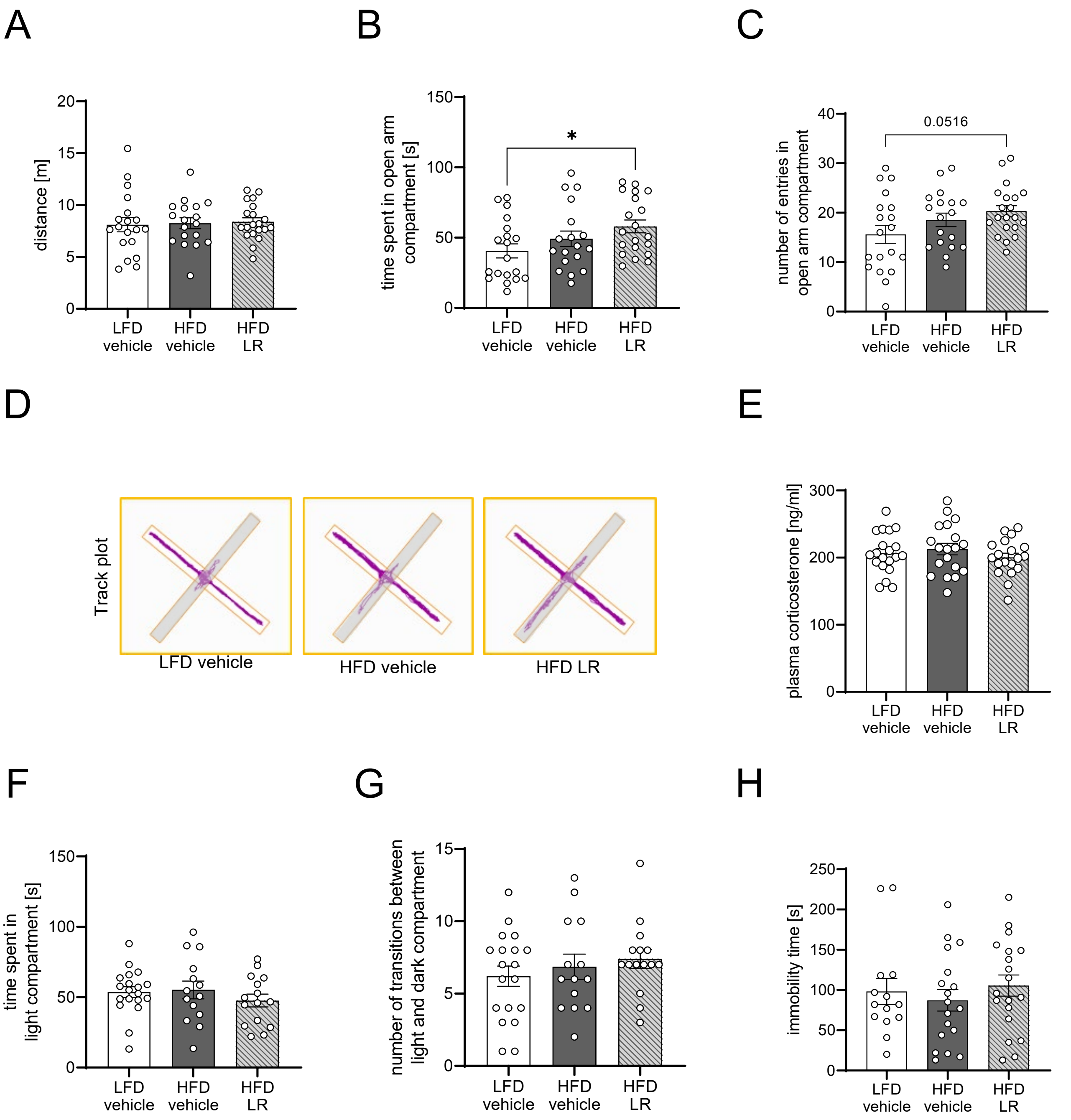

Suppl. Figure 3: LR intervention does not modulate anxiodepressive-like behavior of obese female mice. (A) Distance, **(B)** time spent in the open compartment, and **(C)** number of entries into the open arm compartment of female mice after 10 weeks of HFD during the Elevated Plus Maze (EPM) Test with **(D)** exemplary track plots. Gray area represents the open arm compartment. **(E)** Plasma corticosterone levels of female mice after 10 weeks of HFD after performing the EPM Test. **(F)** Time spent in the light compartment and **(G)** number of transitions between both compartments of female mice after 9 weeks of HFD during the Light/Dark Box

Test. **(H)** Immobility time of female mice after 12 weeks of HFD during the Tail Suspension Test. * $P<0.05$ after 1-way ANOVA with Dunnett's Post-hoc test (n= 14-20). All data are presented as mean±SEM.

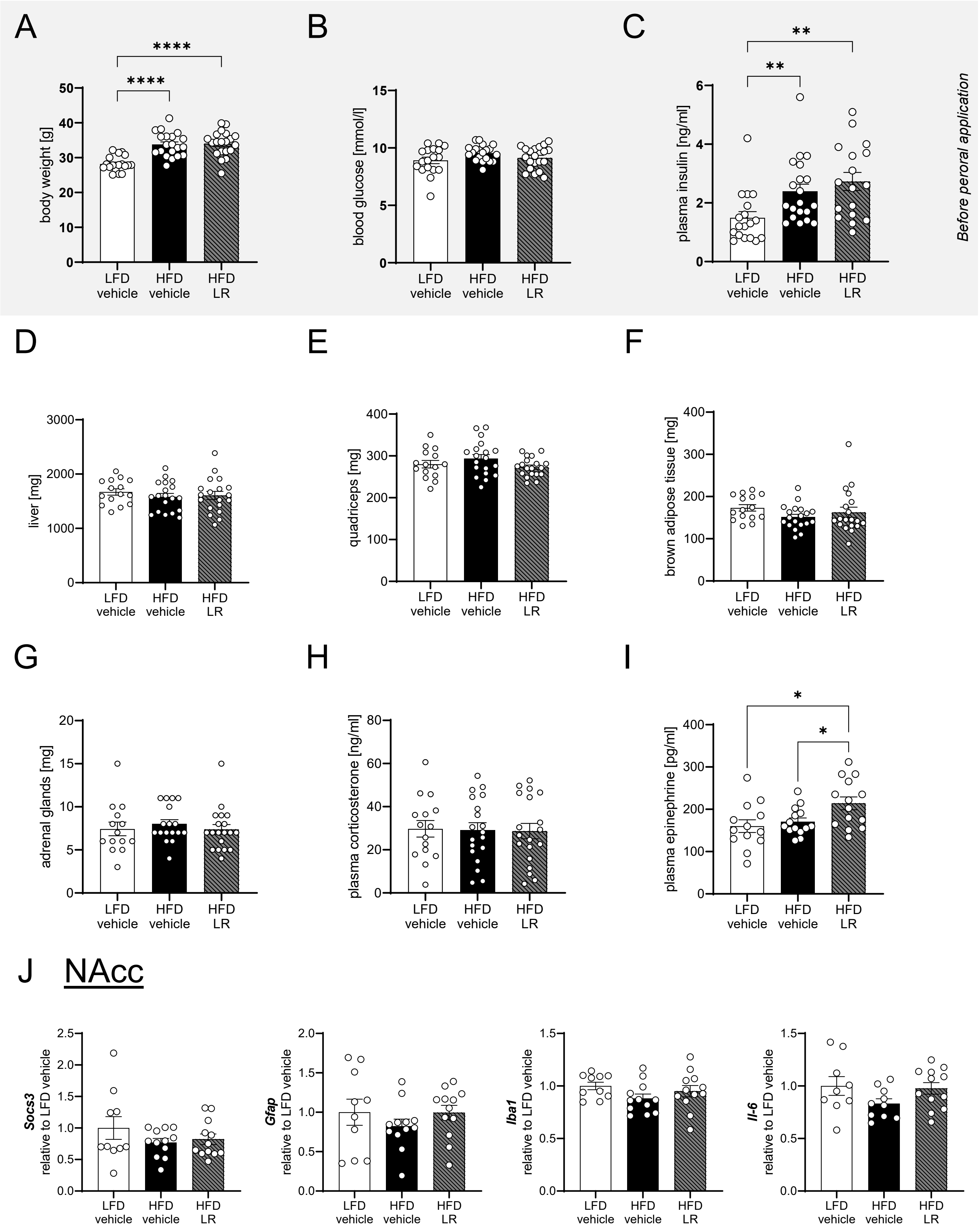
A
B
C
D
E
F
G
H
I
J
NAcc
****
**
*
body weight [g]
blood glucose [mmol/l]
plasma insulin [ng/ml]
Before peroral application
liver [mg]
quadriceps [mg]
brown adipose tissue [mg]
adrenal glands [mg]
plasma corticosterone [ng/ml]
plasma epinephrine [pg/ml]
Socs3 relative to LFD vehicle
Gfap relative to LFD vehicle
Iba1 relative to LFD vehicle
Il-6 relative to LFD vehicle
LFD vehicle
HFD vehicle
HFD LR

Suppl. Figure 4: Additional metabolic data of male mice. (A) Body weight, **(B)** blood glucose, and **(C)** plasma insulin of male mice after 6 weeks of HFD before LR application. **(D)** Liver weight, **(E)** quadriceps weight, **(F)** brown adipose tissue weight, and **(G)** adrenal glands weight of male mice after 13 weeks of HFD. **(H)** Plasma corticosterone and **(I)** plasma epinephrine levels of male mice after 10 weeks of HFD. **(J)** mRNA expression of *Socs3*, *Gfap*, *Iba1*, and *Il-6* in the nucleus accumbens of male mice after 13 weeks of HFD using RT-qPCR. ** P<0.01 and **** P<0.0001 after 1-way ANOVA with Dunnett's Post-hoc test (A,I) or after Kruskal-Wallis test with Dunn's Post-hoc test (C) (n= 15-20). All data are presented as mean±SEM.

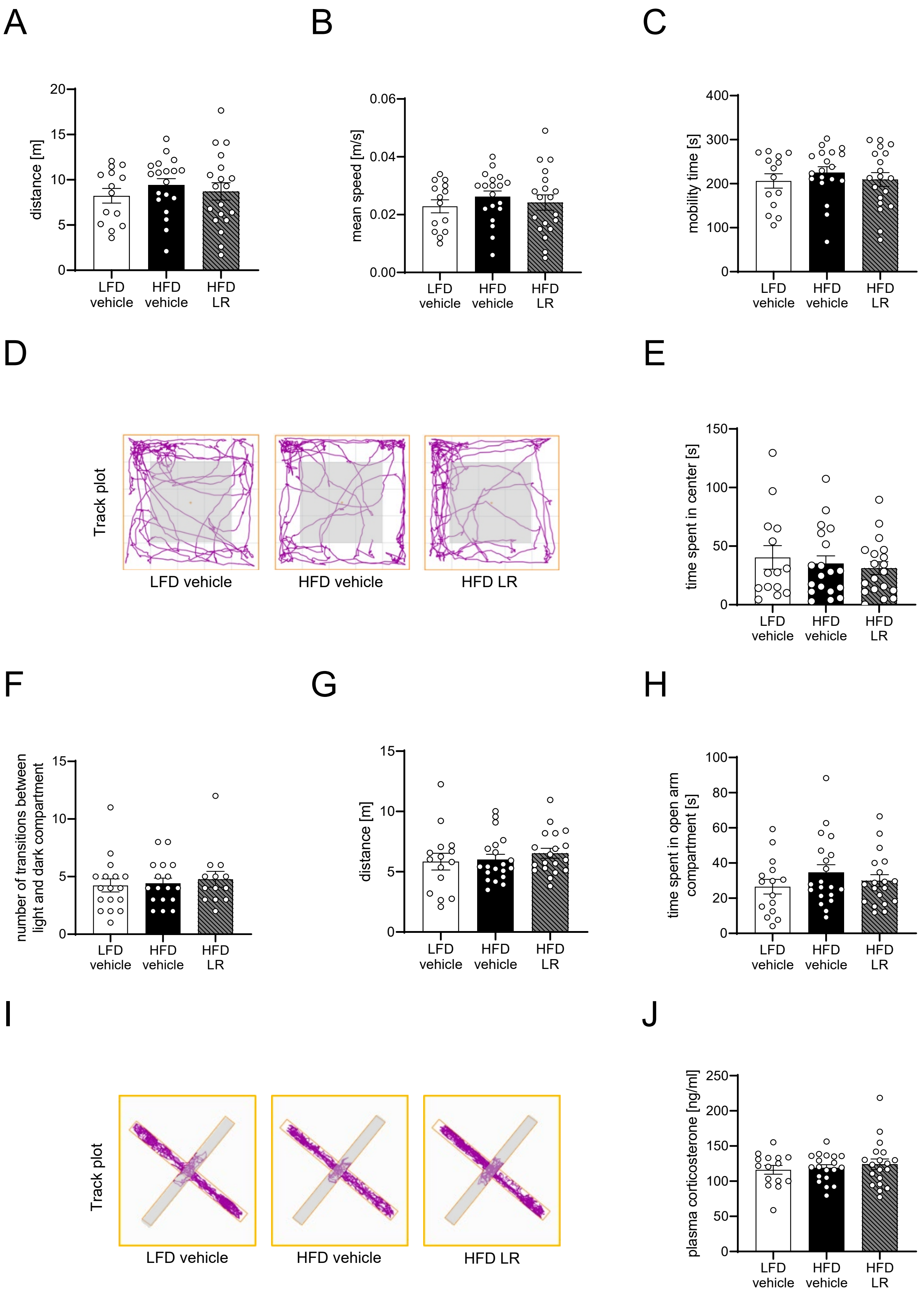
A
distance [m]
B
mean speed [m/s]
C
mobility time [s]
D
Track plot
LFD vehicle
HFD vehicle
HFD LR
E
time spent in center [s]
F
number of transitions between light and dark compartment
G
distance [m]
H
time spent in open arm compartment [s]
I
Track plot
J
plasma corticosterone [ng/ml]
LFD vehicle
HFD vehicle
HFD LR

Suppl. Figure 5: Neither HFD nor LR intervention regulates activity, exploration or stress-induced anxiety in male mice. (A) Distance, **(B)** mean speed, and **(C)** active time of male mice after 8 weeks of HFD during the Open Field Test. **(D)** Exemplary track plots and **(E)** time spent in the center of the field of male mice after 8 weeks of HFD during the Open Field Test. Gray area represents the center of the field. **(F)** Number of transitions between both compartments of male mice after 9 weeks of HFD during the Light/Dark Box Test. **(G)** Distance and **(H)** time spent in the open compartment of male mice after 10 weeks of HFD during the Elevated Plus Maze (EPM) Test with **(I)** exemplary track plots. Gray area represents the open arm compartment. **(J)** Plasma corticosterone levels of male mice after 10 weeks of HFD after performing the EPM Test. n= 14-20. All data are presented as mean±SEM.

A

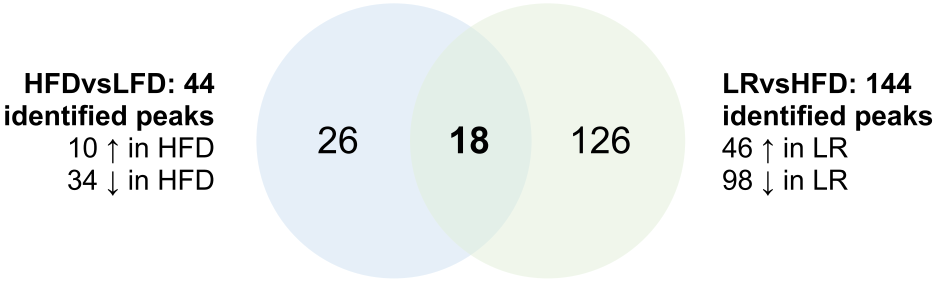

B

CSF: HFD vehicle vs. LFD vehicle

MAP_ID	MAP_NAME	N_metab	N_mapped	N_significant	N_mapped_significant	Fisher_Test_p_value
map04742	Taste transduction	150	3	10	2	0.0116452022492291

CSF: HFD LR vs. HFD vehicle

MAP_ID	MAP_NAME	N_metab	N_mapped	N_significant	N_mapped_significant	Fisher_Test_p_value
map00564	Glycerophospholipid metabolism	150	4	12	2	0.0323170835539004
map00020	Citrate cycle (TCA cycle)	150	6	12	2	0.0736959684948187
map00720	Carbon fixation pathways in prokaryotes	150	6	12	2	0.0736959684948187

C

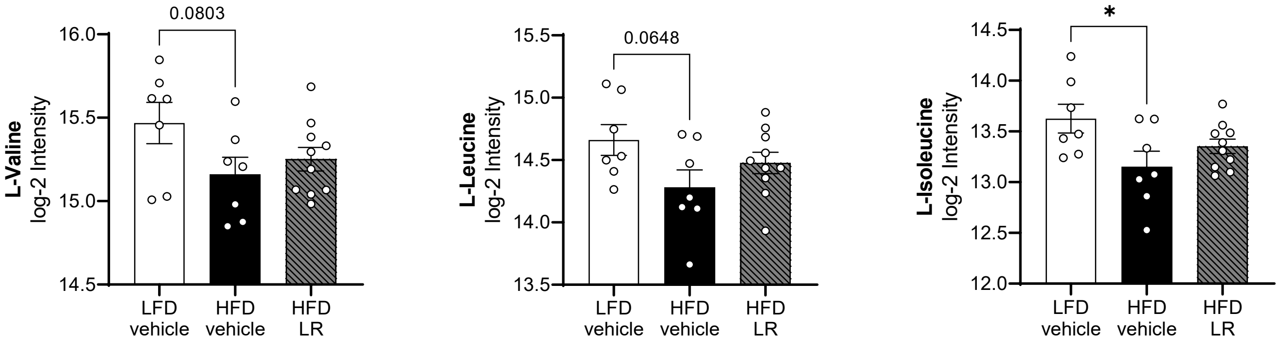

D

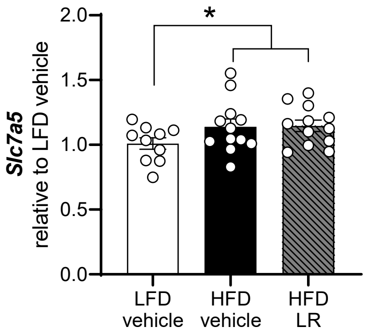

Suppl. Figure 6: HFD drives changes in abundance of branched chain amino acids in the CSF of male mice. (A) Visualization of significant annotated and non-annotated CSF metabolites within and between both comparisons ('diet effect' and 'lactobacillus effect') of male mice after 13 weeks of HFD. **(B)** KEGG pathway analysis of annotated CSF metabolites and fatty acids of male mice after 13 weeks of HFD (adjPvalue<0.05 after Fisher's Exact test). **(C)** Relative abundance of L-Valin, L-Leucin, and L-Isoleucin in the CSF of male mice after 13 weeks of HFD. **(D)** mRNA expression of *Slc7a5* in the nucleus accumbens of male mice after 13 weeks of HFD using RT-qPCR. * P<0.05 after 1-way ANOVA with Dunnett's Post-hoc test (n= 7-10) or * P<0.05 after unpaired two-tailed Student's t-test. All data are presented as mean±SEM. CSF: cerebrospinal fluid. ↑: significantly more abundant, ↓: significantly less abundant.

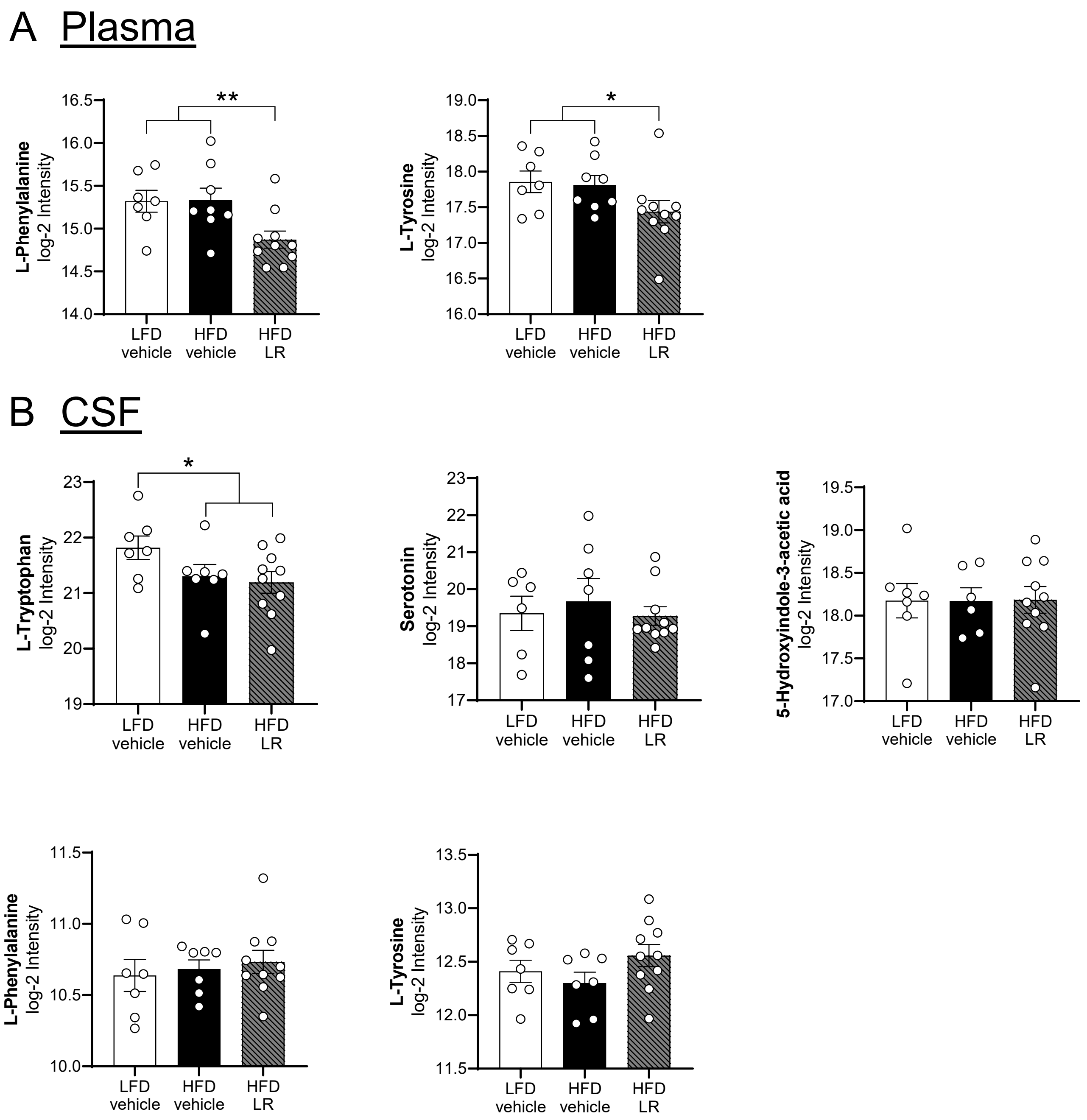

Suppl. Figure 7: High-fat diet and LR application cause mild alterations in dopaminergic and serotonergic metabolites in male mice. (A) Relative abundance of L-Tryptophan, L-Phenylalanine, and L-Tyrosine in plasma. **(B)** Relative abundance of L-Tryptophan, Serotonin, 5-Hydroxylindole-3-acetic acid, L-Phenylalanine, and L-Tyrosine in CSF. * P<0.05 and ** P<0.01 after unpaired two-tailed Student’s t-test. All data are presented as mean±SEM.

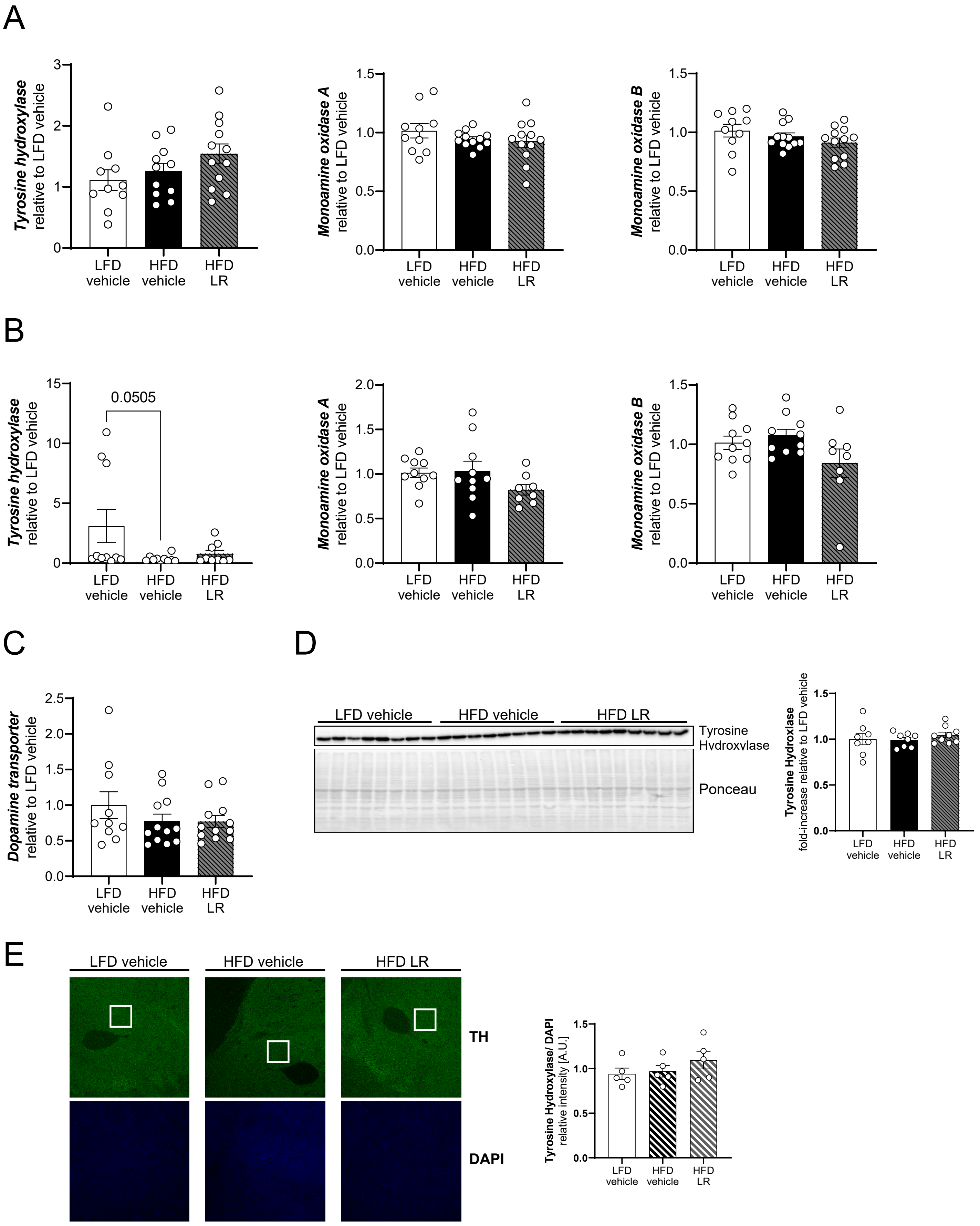
A
Tyrosine hydroxylase relative to LFD vehicle
Monoamine oxidase A relative to LFD vehicle
Monoamine oxidase B relative to LFD vehicle
LFD vehicle
HFD vehicle
HFD LR
B
0.0505
C
Dopamine transporter relative to LFD vehicle
D
LFD vehicle
HFD vehicle
HFD LR
Tyrosine Hydroxylase
Ponceau
Tyrosine Hydroxlase fold-increase relative to LFD vehicle
E
TH
DAPI
Tyrosine Hydroxylase/ DAPI relative intensity [A.U.]

Suppl. Figure 8: Both HFD and LR do not regulate tyrosine hydroxylase protein expression in the nucleus accumbens of male mice. (A) mRNA expression of tyrosine hydroxylase (TH) and monoamine oxidase A/B in the caudate putamen and **(B)** VTA/SN of male mice after 13 weeks of HFD (n= 9-12). **(C)** mRNA expression of Dopamine transporter in the nucleus accumbens of male mice after 13 weeks of HFD. **(D)** Protein expression of tyrosine hydroxylase in the nucleus accumbens of male mice after 13 weeks of HFD using western blot (n= 8-9). **(E)** Protein expression of tyrosine hydroxylase (TH) in the nucleus accumbens of male mice after 14 weeks of HFD using immunohistochemistry (n= 5). White box indicates the area of analysis. * $P<0.05$ after 1-way ANOVA with Dunnett's Post-hoc test (B). All data are presented as mean±SEM.

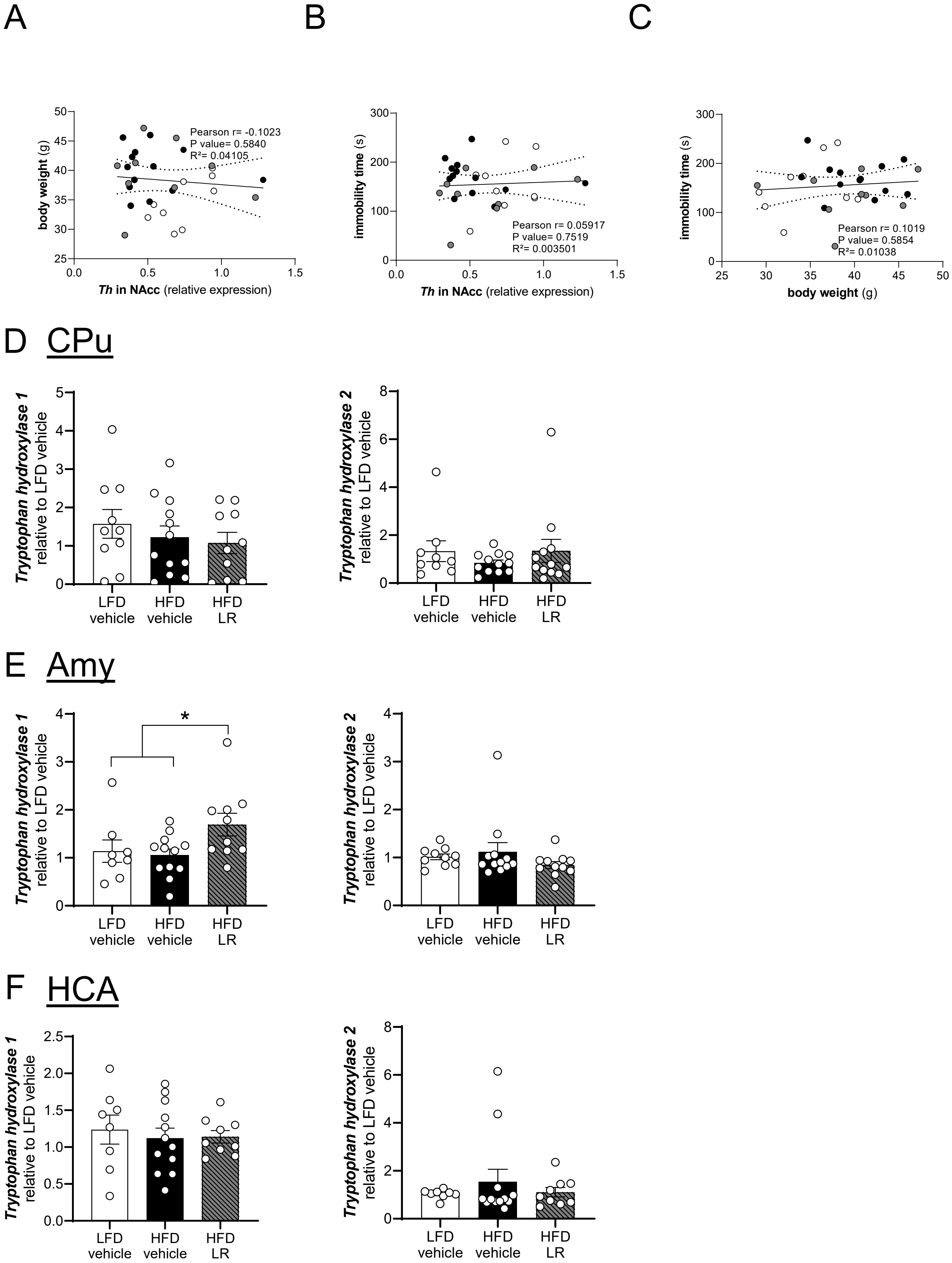
A
B
C
body weight (g)
Th in NAcc (relative expression)
Pearson r= -0.1023
P value= 0.5840
R²= 0.04105
immobility time (s)
Pearson r= 0.05917
P value= 0.7519
R²= 0.003501
body weight (g)
Pearson r= 0.1019
P value= 0.5854
R²= 0.01038
D CPu
Tryptophan hydroxylase 1
relative to LFD vehicle
Tryptophan hydroxylase 2
relative to LFD vehicle
LFD vehicle
HFD vehicle
HFD LR
E Amy
*
F HCA

Suppl. Figure 9: Gene expression of tryptophan hydroxylase remains largely unaltered in male mice after 13 weeks of HFD. (A) Correlation analysis of body weight and relative gene expression of *Th*, **(B)** immobility time during the Tail Suspension Test (TST) and relative gene expression of *Th*, and **(C)** immobility time during the TST and body weight. All data are presented relative to *Tbp* ($2^{\Delta CT}$). Continuous line represents the mean and the dotted line represents the error after linear regression analysis. **(D)** mRNA expression of Tryptophan hydroxylase 1 and 2 in caudate putamen (CPu), **(E)** amygdala (Amy), and **(F)** hippocampus (HCA). * $P<0.05$ after unpaired two-tailed Student's t-test. All data are presented as mean±SEM.

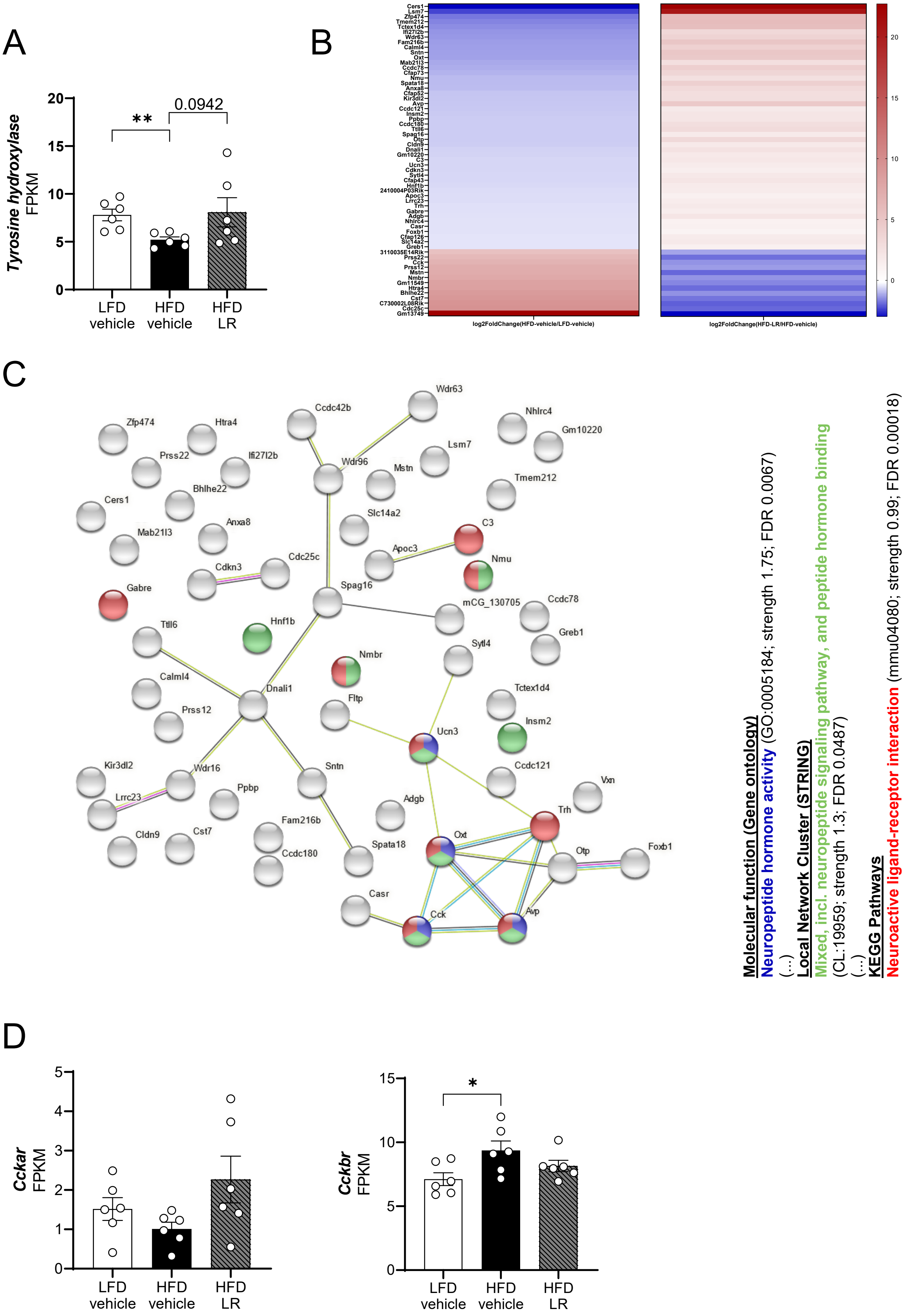
A
B
C
D
Tyrosine hydroxylase
FPKM
**
0.0942
LFD vehicle
HFD vehicle
HFD LR
log2FoldChange(HFD-vehicle/LFD-vehicle)
log2FoldChange(HFD-LR/HFD-vehicle)
Molecular function (Gene ontology)
Neuropeptide hormone activity (GO:0005184; strength 1.75; FDR 0.0067)
(...)
Local Network Cluster (STRING)
Mixed, incl. neuropeptide signaling pathway, and peptide hormone binding
(CL:19959; strength 1.3; FDR 0.0487)
(...)
KEGG Pathways
Neuroactive ligand-receptor interaction (mmu04080; strength 0.99; FDR 0.00018)
Cckar
FPKM
Cckbr
FPKM
*

Suppl. Figure 10: RNA sequencing analysis of the nucleus accumbens of male mice after 13 weeks of HFD. (A) Tyrosine hydroxylase mRNA levels using RNA sequencing (n= 6). **(B)** log2FoldChange of 61 common differentially expressed genes (DEGs) between LFD and HFD (left panel) as well as HFD and LR (right panel) in nucleus accumbens of male mice. log2 Fold Change> |1.00| and Pvalue<0.05; red: upregulated; blue: downregulated. **(C)** 58 of 61 common differentially expressed genes (DEGs) between LFD and HFD as well as HFD and LR were mapped to proteins in mus musculus and used for protein network analysis and subsequent pathway analyses. blue: Neuropeptide hormone activity; green: Mixed, incl. neuropeptide signaling pathway, and peptide hormone binding; red: Neuroactive ligand-receptor interaction; GO: gene ontology; FDR: false discovery rate (adjusted P value). **(D)** RNA sequencing analysis of CCK receptors A and B (n= 6). * P<0.05 after 1-way ANOVA with Dunnett's Post-hoc test (D) and ** P<0.01 after unpaired two-tailed Student's t-test for each comparison (A). All data are presented as mean±SEM.

Supplementary methods

Animals

Study design

Three-week-old female and male C57BL/6N wildtype mice (strain code: 027) were obtained from Charles River Laboratories (Sulzfeld, Germany) and group-housed in a temperature-controlled room (22 ± 1 °C) on a 12-hour light/12-hour dark cycle with free access to food and water. For the high fat diet intervention (HFD, 45 % of calories from fat, #D12451, Research Diets, Inc.), HFD pellets were gradually added to the LFD (low fat diet, 10 % of kcal from fat, #D12450H, Research Diets, Inc.) over the course of two weeks, starting at week four of age, to allow adaptation of the gastrointestinal tract to the new diet. The difference in calories between these diets was only based on the fat content, while the diets were matched for remaining macronutrients. Sampling of blood, cerebrospinal fluid (CSF), and organs were carried out at week 19 of age. In an independent behavior cohort using male mice, organ collection was carried out at week 20 of age to perform the Splash Test at week 19 of age. All animal study and care protocols were in accordance with the animal welfare committees of the German Institute of Human Nutrition and approved by the local authorities (State Agency of Environment, Health and Consumer Protection, LUGV, Brandenburg, Germany) (TVA 2347-38-2019).

To investigate the effect of LR application during obesity development, male C57BL/6J mice were obtained from Jackson Laboratories at the age of 6 weeks (previously described in [1]). Briefly, mice were changed from a chow diet to a compositionally defined low fat diet (LFD) for a 12-day acclimatization. Afterwards, mice were either continuously fed with the LFD or received a high fat diet (HFD, 45 % of calories from fat, Ssniff Spezialdiäten, corresponds to #D12451). Within those 12 weeks of experimental feeding, mice were orally gavaged daily. After 12 weeks, the mice were anaesthetized with isoflurane (Fresenius Kabi), killed by cervical dislocation, and organs were collected. All experiments conducted in accordance with the Canadian Council on Animal Care guidelines and regulations approved by Laval University, Canada (license 2017-086-1).

Peroral application

Lactobacillus rhamnosus (Lacticaseibacillus rhamnosus 0030, here: LR) was prepared and provided by Organobalance GmbH, Berlin. From week 12 of age until the end of experiments, all mice received a daily peroral gavage of either 100 µl vehicle (phosphate-buffered saline, PBS, Gibco) or 100 µl LR ($1x10^8$ CFU of LR in PBS) to ensure accurate dosage. On days when behavioral tests were performed, intragastric gavage was paused.

Basic metabolic phenotyping

Body weight was monitored weekly. Blood glucose was measured using a Glucometer Contour XT (Bayer, Germany). Plasma insulin was measured with an ELISA from Alpco (Alpco Salem, BioCat GmbH, Switzerland). Plasma leptin was measured using an ELISA from R&D Systems (R&D Systems/ Bio-Techne GmbH, Germany), and plasma corticosterone levels were determined using an ELISA from Enzo (Enzo Life Sciences GmbH, Germany). Plasma epinephrine levels were quantified using an ELISA from Biomatik (Biomatik Corporation, BioCat GmbH, Germany). All samples were collected from random fed mice.

Behavioral assessment

For the Open Field Test (OFT) the mouse was placed into a 50 cm x 50 cm open box and spontaneous activity was recorded for six minutes using the ANY-maze Video Tracking System (Stoelting Co., Wood Dale, IL, United States). Mice who traveled less than 2 m during the test were excluded from analysis as they did not show spontaneous exploration of a novel environment. For the Light/Dark Box Test (LDB), the mouse was placed into a 40 cm x 40 cm box which consists of a dark (20 cm x 40 cm, opaque/ closed) and a light compartment (20 cm x 40 cm, clear/ open). The mouse was recorded for six minutes using the ANY-maze Video Tracking System and time spent in the light compartment as well as number of transitions between compartments were analyzed. Mice who spent more than 359 sec in the light compartment were excluded from analysis as they did not exhibit natural aversion of a brightly illuminated novel environment and thereby 'normal' behavior. The Elevated Plus Maze Test (EPM), or Elevated X Maze Test, was additionally used to evaluate stress-induced anxiety and exploration. The elevated apparatus (50 cm) consisted of two open (length x width x height; 30 cm x 5 cm x 0.5 cm) and two closed arms (opaque walls, 30 cm x 5 cm x 15

cm) which met at a center platform (5 cm x 5 cm x 0.5 cm). 24 h prior to the behavior test (baseline) as well as immediately after the test blood was sampled to measure plasma corticosterone. The mouse was recorded for ten minutes using the ANY-maze Video Tracking System. After the second blood sampling, the mouse was returned to its home cage. In the Tail Suspension Test (TST), the mouse was fixed at its tail and recorded for six minutes. The analysis for this test was performed manually. Video analysis was performed blinded until immobility time of all animals was assessed. In the Splash Test, the mouse was placed in an open cage and a 10 % sucrose solution was applied onto the dorsal coat of the mouse. For five minutes, the mouse was recorded using a camera to subsequently analyze latency to groom as a marker for lack of motivation. The analysis for this test was performed manually. Video analysis was performed blinded until grooming latency of all animals was assessed.

Final procedures

Random fed mice were deeply anesthetized by i.p. injection of Ketamine (100 mg/kg body weight)/ Xylazine (16 mg/kg body weight) and were killed by heart puncture and cervical dislocation followed by dissection of peripheral organs (approx. 16 h – 20 h after the last peroral gavage). Brain areas were dissected using mouse brain matrix (Zivic Instruments, Pittsburgh, PA, United States). Of note, ventral tegmental area and substantia nigra were pooled as one sample and the nucleus accumbens sample is an enrichment of the core region. Tissues were snap-frozen in liquid nitrogen and stored at -80 °C until further processing. Cerebrospinal fluid (CSF) was collected using the cisterna magna puncture technique [2, 3]. After collection, the CSF was stored at -80 °C.

Intra-cardiac perfusion

For intra-cardiac perfusion, mice were deeply anesthetized by i.p. injection of Ketamine (100 mg/kg body weight)/Medetomidine (0.8 mg/kg body weight). The thorax was opened to expose the heart and to inject a 21 G cannula (Venofix®, B.Braun) into the left ventricle. The right ventricle of the heart was opened and 0.9 % NaCl-solution was steadily injected (10 ml/min) for about 2 min until the fluid exiting the heart ran clear. To fix the tissue, 4 % PFA (paraformaldehyde) solution was steadily injected for about 5 min. Afterwards, the brain and peripheral tissues were collected and stored in 4 % PFA until further processing.

Brain sectioning

After intra-cardiac perfusion, brains were immersed with 4 % PFA at 4 °C overnight and following immersed with 20 % sucrose in 0.02 M KPBS (potassium phosphate buffered saline), pH 7.4 for 24 h at 4 °C. Brains were carefully frozen before storage at -80 °C. Brains were coronally sectioned at a thickness of 30 µm using a sledge microtome (pfm medical, Cologne, Germany). Brain sections were collected and stored in 'anti-freeze medium' (100 ml 10X PBS, 400 ml ddH2O, 300 ml ethylene glycol, 200 ml glycerol) at -20 °C until staining.

Immunohistochemistry

This protocol was similarly described in [4]. Free-floating sections were rinsed with 0.02 M KPBS (3 times for 10 min), incubated in 0.3 % glycine in 0.02 M KPBS for 10 min, rinsed with 0.02 M KPBS for 5 min, and incubated in 0.03 % SDS in 0.02 M KPBS for 10 min. Afterwards, free-floating sections were blocked for 1 h in 0.25 % Triton-X with 3 % goat serum in 0.02 M KPBS at RT and then incubated with the primary antibody in SignalStain® Antibody Diluent (#8112, Cell Signaling, Cambridge, United Kingdom) overnight at 4 °C. The next day, sections were rinsed with 0.02 M KPBS (once for 5 min, 3 times for 20 min) and following incubated with fluorescence-conjugated secondary antibody for 1 h at RT. The stained free-floating sections were rinsed with 0.02 M KPBS (once for 5 min, 3 times for 20 min) and then mounted onto Superfrost Plus microscope slides (VWR International GmbH) in ddH_2O. Excess ddH_2O from adhered sections was carefully removed. Slides were dried at room temperature before VECTASHIELD Antifade Mounting Medium with DAPI (Vector Laboratories, Inc., Burlingame, CA, United States) was applied to the slides before placing the coverslip. The slides were left to cure overnight before sealing with nail polish the next day. Sections were imaged with a 10X objective using confocal microscopy (LSM 780 from Carl ZEISS, Oberkochen, Germany) and the analysis was performed using ZEN software (version 2.3) from ZEISS. The primary antibody was rabbit anti-Tyrosine Hydroxylase (1:500; AB152; Merck, Darmstadt, Germany). The fluorescence-conjugated secondary antibody was goat anti-rabbit AlexaFluor488 (1:500 in 0.02 M KPBS with 0.25 % Triton-X; ab150077; Abcam, Cambridge, United Kingdom).

Molecular analyses

RNA isolation

Total RNA from tissue was extracted using ReliaPrep RNA Tissue Miniprep System (Promega, Walldorf, Germany) according to manufacturer's instructions, including DNAse I treatment. RNA concentration and quality of the samples was determined using the NanoDrop^{TM} Spectrophotometer (Thermo Fisher Scientific).

Quantitative real-time PCR

Between 100 ng and 1 µg RNA were reverse-transcribed using oligo(dT)15 primers (Promega), random hexameric primers (Promega), Thermo Fisher Scientific dNTP-Set, and M-MLV reverse transcriptase (Promega). The quantitative real-time polymerase chain reaction (RT-qPCR) was performed with 10 ng (= 2 µl) cDNA, 5 µl GoTaq 1-Step RT-qPCR System mix (Promega), and 200 nM of each forward and reverse primer (obtained from Sigma-Aldrich/Merck, see Supplementary Table S1) per well. Fluorescence was monitored using the ViiA 7 Real-Time PCR System (Applied Biosystems^{TM}, Thermo Fisher Scientific). Each run was followed by a melt curve (90 °C to 60 °C) for quality control. Relative quantification of gene expression levels was performed according to the ΔΔCT method using TATA-box-binding protein (*Tbp*) as reference gene. Data were expressed as $2^{\Delta\Delta CT}$ and relative to the respective control group, if not stated otherwise.

Transcriptome analysis

RNA sequencing was performed on a subset of RNA samples from the nucleus accumbens (n= 6 per group). RNA quality control was performed with the Agilent 2100 Bioanalyzer (minimal RNA integrity: 7.0) before library construction. Sequencing analysis was performed by BGI Genome Sequencing Service in Hongkong on DNBSEQ (DNBSEQ Technology) platform. Briefly, adapters and low-quality reads were filtered and removed after transcriptome sequencing. Reads were aligned to the reference genome(mm10) using HISAT2 (Hierarchical Indexing for Spliced Alignment of Transcripts). For gene expression analysis, clean reads were mapped to reference using Bowtie2, and gene expression level was calculated with RSEM. Finally, differentially expressed genes (DEGs) were detected (LFD vs HFD, HFD vs LR) with the following parameters: log2-fold change [log2FC]>|1.00| and raw Pvalue<0.05). Novel transcripts ('BGI_novel') were excluded from analysis. The raw data are openly

available in GEO (reference number: GSE228484). Following, a descriptive analysis of DEGs from LFD vs HFD and HFD vs LR was performed and DEGs were compared and displayed using VENNY2.1 (https://bioinfogp.cnb.csic.es/tools/ venny/index.html) to identify common DEGs. Additionally, gene symbols were submitted to STRING: functional protein association networks (Version 11.5), gene symbols were matched to proteins in mus musculus. 58 out of 61 DEGs were successfully mapped to proteins and then were used for network building. Next, different pathway analyses were conducted within the network and functional enrichment analyses (Gene Ontology analysis and KEGG pathway) was performed within the network using Adjusted Pval (FDR: false discovery rate), calculated with Benjamini-Hochberg procedure, as threshold, which were confirmed using the PANTHER (Protein ANalysis THrough Evolutionary Relationships) Classification System V16.0.

Protein isolation, SDS-PAGE, and western blot

Brain tissue was homogenized in 50 µl RIPA lysis buffer using a pestle mixer (VWR Inter-national GmbH, Radnor, PA, United States), incubated for 30 min on ice and subsequently centrifuged at 13.000 RPM for 30 min at 4 °C. Isolated proteins were quantified using Pierce^{TM} 660 nm Protein Assay Reagent (Thermo Scientific). 4X LSB with 10 % β-mercaptoethanol was added to the protein samples (15 µg of protein per sample) and adjusted with RIPA lysis buffer to final volume, followed by heat-denaturation for 5 min at 95 °C. Samples were loaded onto a 5 % stacking gel to allow proper protein migration towards the 10 % separating gel. Separation in an electrical field was carried out at 200 V using the Mini-PROTEAN Tetra Cell (Bio-Rad Laboratories, Inc, Hercules, CA, United States). Proteins were transferred for 3 h at 90 V to a polyvinylidene difluoride (PVDF) (GE HealthCare Life Science, Amersham, United Kingdom) membrane using a wet blot transfer cell system (Criterion^{TM} Blotter/ Mini Trans-Blot Cell, Bio-Rad). After the Ponceau staining, membranes were blocked in StartingBlock^{TM} T20 Blocking Buffer (Thermo Scientific) for 1 h, and following incubated with a primary antibody dilution overnight at 4 °C (Tyrosine Hydroxylase, ab112, Abcam, 1:1.000 in 5 % BSA in 1X TBS-T). Afterwards, membranes were washed 3 times for 5 min with 1X TBS-T (tris buffered saline, 10 mM Tris, 150 mM NaCl, 0.05 % Tween 20) before incubating with a secondary antibody dilution for 1 h at RT (HRP-conjugated goat anti-rabbit, #7074, Cell Signaling, Cambridge, United Kingdom, 1:2.000 in 5 % BSA in 1X TBS-T). Before imaging, membranes were washed minimum 4 times for 5 min with 1X TBS-T. For visualization, membranes were

incubated with Pierce™ ECL Western Blotting Substrate (Thermo Scientific) for 1 min and the signal was detected with the ChemiDoc Touch Imaging System (Bio-Rad). Ponceau staining was used as loading control for normalization to total protein content using Image Lab 5.1 software (Bio-Rad).

Untargeted metabolomics and lipidomics

Both CSF and plasma samples were subjected to an untargeted approach of metabolome and lipidome analysis. Sample processing, measurements and bioinformatics analysis were performed by metaSysX, Potsdam-Golm, Germany. Briefly, sample preparation was performed according to metaSysX standard procedure (modified from [5]; method information provided by metaSysX). 10 µl of CSF and 50 µl of plasma were used for MTBE (Methyl-tert-Butyl Ether) two phase extraction to separate the lipid fraction. For identification of hydrophilic and lipophilic compounds, samples were measured with a Waters ACQUITY Reversed Phase Ultra Performance Liquid Chromatography (RP-UPLC) coupled to a Thermo-Fisher Exactive mass spectrometer. For identification of volatilized compounds, samples were measured on an Agilent Technologies GC coupled to a Leco Pegasus HT mass spectrometer which consists of an EI ionization source and a TOF mass analyzer. Compound annotation was performed based on mass-to-charge ratio and the retention time. Metabolites with a retention time (retention index for GC analysis [6]) and a mass-to-charge ratio that did not result in a match in the in-house metaSysX database or Fiehn library were kept as not assigned metabolites. Data normalization was performed for each platform separately for the median of intensities of each sample to the median of the group. Following, data were transformed and displayed as log-2 intensity. Additionally, KEGG Pathway Enrichment Analysis (PAE) was performed by metaSysX. PAE was performed on the CSF and plasma datasets separately. Only metabolites mapped to KEGG, i.e. they have a KEGG identifier, were considered. In total, 150 and 161 metabolites were mapped in CSF and plasma, which cover 159 and 172 pathways according to the KEGG database. For every t-test comparison, for example LFD vehicle vs HFD vehicle, a metabolite was declared significant if $p<0.05$. Of note, the raw non-corrected p-value was used. For every pathway, for example fatty acid metabolism, and for each t-test comparison, a contingency matrix was computed which allowed to perform a Fisher's exact test.

DNA preparation and 16S amplicon sequencing

Sample processing and all bioinformatics analysis were performed and provided by Organobalance GmbH, and was previously described in [1]. Briefly, bacterial DNA from cecal samples (intestinal content) was extracted using a NucleoSpin soil kit (Macherey-Nagel, Düren, Germany) according to manufacturer's instructions (semiautomated protocol). Prior to DNA extraction all samples were heat inactivated for 15 min at 95 °C. 16S rRNA gene amplification targeting the V3-V4 region was performed using forward primer 341-2FDI (5'-ACACTCTTTCCCTACACGACGCTCTTCCGATCTCCTA CGGGNGGCWGCAG-3') and reverse primer 805.2RDI (5'-AGACGTGTG CTCTTCCGATCTGACTACHVGGGTATCTA ATCC-3'). 16S rRNA gene amplicons were indexed using Nextera XT index kit v2 (Illumina, San Diego, CA, United States). Libraries were cleaned using Agencourt AMPure XP beads (Beckman Coulter, Brea, CA, United States) and sequenced using an Illumina MiSeq desk-top sequencer using the MiSeq Reagent Kit V3 (Illumina) for 2 × 300 bp paired-end. The generation of an amplicon sequence variant (ASV) table was done with usearch version 10.0.240.

Bioinformatics processing, OTU clustering and classification

The generation of Operative Taxonomic Unit (OTU) tables was done with usearch version 10.0.240 [7]. Primer binding regions were removed with fastx_truncate and reads were filtered to contain less than one error per read. The quality filtered reads were denoised with unoise3. Otu abundance was calculated by mapping with usearch_global using a 97 % identity threshold. Taxonomical classification was done with the qiime naive bayes classifier trained on the Arb Silva database. The phylogenetic tree was made by aligning the 16S sequences with mafft and the tree was inferred by FastTree. The bash scripts running the pipeline is deposited in https://gitlab.nzcorp.net/bioinf/bioinf_utils/-/blob/master/src/RTEC2001067/ nzpipeline/edgarAndQiime.bash.

Beta diversity was analyzed by calculating Unifrac distances, generating the two-dimensional Principal Coordinate Analysis plots (PCA). Variance analysis of beta diversity was done with adonis (Permutational multivariate analysis) from the vegan package. Rarefied count data (minimum sequence depth 5000) were used for the beta diversity analysis. The R package microbiomeutils was used as a basis for the statistical analysis of the microbiome data. The Bioconductor package DESeq2 [8] for detecting species in differential abundance was used for the differential abundance

analysis. The relative abundance associated with the DESeq2 tables are mean relative abundances. These values are not a part of the DESeq2 output and are not used to calculate the DESeq2 reported log2 fold changes. The raw (non-rarefied) count data were used for the DESeq2 analysis.

Supplementary tables

Target gene	Forward sequence	Reverse sequence
Tbp (TATA box binding protein)	CTGGAATTGTACCGCAGCTT	ATGATGACTGCAGCAAATCG
Cck (Cholecystokinin)	ACTGCTAGCGCGATACATCC	CCCACTACGATGGGTATTCG
Cckar (Cholecystokinin A receptor)	GACAGCCTTCTTATGAATGGGAG	GCTGAGGTTGATCCAGGCAG
Cckbr (Cholecystokinin B receptor)	GATGGCTGCTACGTGCAACT	CGCACCACCCGCTTCTTAG
Dat (Dopamine transporter)	GGAGACCTGGAGCAAGAAAATC	GCAATAACCATGAAGAGCAGG
Drd2 (Dopamine receptor 2)	CCACTCAAGGGCAACTG	TGACAGCATCTCCATTTCCAG
Drd4 (Dopamine receptor 4)	GAAGGAGAGGCGCCAAGAT	GACTACCACCGGCAGGACTC
Gfap (Glial fibrillary acidic protein)	CGTGTGGATTTGGAGAGAAAG	GTGAGGTCTGCAAACTTAGACC
Iba1 (Ionized calcium binding adaptor molecule 1)	CGATGATCCCAAATACAGCAATG	CCCAAGTTTCTCCAGCATTC
Il-6 (Interleukin 6)	CAAAGCCAGAGTCCTTCAGAG	GTCCTTAGCCACTCCTTCTG
MaoA (Monoamine oxidase A)	TGGTTCTTGTGGTATGTGAGG	AGCTTCACTTTATCCCCAAGG
MaoB (Monoamine oxidase B)	GCCCGTCCATTATGAAGAGAAG	CTGTTTCAGTGCCTGCAAAG
Slc7a5 (Solute carrier family 7 member 5)	CTTCGGCTCTGTCAATGGGT	TTCACCTTGATGGGACGCTC
Socs3 (Suppressor of cytokine signaling 3)	CCTATGAGAAAGTGACCCAGC	TTTGTGCTTGTGCCATGTG
Th (Tyrosine hydroxylase)	AAGATCAAACCTACCAGCCG	TACGGGTCAAACTTCACAGAG
Tph1 (Tryptophan hydroxylase 1)	AACAAAGACCATTCCTCCGA	TGTAACAGGCTCACATGATT
Tph2 (Tryptophan hydroxylase 2)	TCGAAATCTTCGTGGACTGC	CGGATTCAGGGTCACAATG

Supplementary Table S1

Supplementary references

1. Larsen, I.S., et al., *Fungal lysozyme leverages the gut microbiota to curb DSS-induced colitis.* Gut Microbes, 2021. **13**(1): p. 1988836.
2. Lim, N.K., et al., *An Improved Method for Collection of Cerebrospinal Fluid from Anesthetized Mice.* J Vis Exp, 2018(133).
3. Liu, L. and K. Duff, *A technique for serial collection of cerebrospinal fluid from the cisterna magna in mouse.* J Vis Exp, 2008(21).
4. Lippert, R.N., et al., *Maternal high-fat diet during lactation reprograms the dopaminergic circuitry in mice.* J Clin Invest, 2020. **130**(7): p. 3761-3776.
5. Salem, M.A., et al., *Protocol: a fast, comprehensive and reproducible one-step extraction method for the rapid preparation of polar and semi-polar metabolites, lipids, proteins, starch and cell wall polymers from a single sample.* Plant Methods, 2016. **12**: p. 45.
6. Cuadros-Inostroza, A., et al., *TargetSearch--a Bioconductor package for the efficient preprocessing of GC-MS metabolite profiling data.* BMC Bioinformatics, 2009. **10**: p. 428.
7. Edgar, R.C., *UNOISE2: improved error-correction for Illumina 16S and ITS amplicon sequencing.* bioRxiv, 2016: p. 081257.
8. Love, M.I., W. Huber, and S. Anders, *Moderated estimation of fold change and dispersion for RNA-seq data with DESeq2.* Genome Biol, 2014. **15**(12): p. 550.
